# The gut microbiota metabolite urolithin A inhibits NF-κB activation in LPS stimulated BMDMs

**DOI:** 10.1038/s41598-021-86514-6

**Published:** 2021-03-29

**Authors:** Khalid N. M. Abdelazeem, M. Zaher Kalo, Sandra Beer-Hammer, Florian Lang

**Affiliations:** 1 Department of Internal Medicine III, Eberhard Karls University of Tübingen, Tübingen, Germany; 2 Radiation Biology Research Department, National Centre for Radiation Research and Technology, Atomic Energy Authority, Cairo, Egypt; 3 Department of Pharmacology, Experimental Therapy and Toxicology, Institute of Experimental and Clinical Pharmacology and Pharmacogenomic, University of Tübingen, Wilhelmstrasse 56, 72074 Tübingen, Germany

**Keywords:** Immunology, Pathogenesis

## Abstract

Inflammation is a natural defense process of the innate immune system, associated with the release of proinflammatory cytokines such as interleukin-1β, interleukin-6, interleukin-12 and TNFα; and enzymes including iNOS through the activation and nuclear translocation of NF-κB p65 due to the phosphorylation of IκBα. Regulation of intracellular Ca^2+^ is considered a promising strategy for the prevention of reactive oxygen species (ROS) production and accumulation of DNA double strand breaks (DSBs) that occurs in inflammatory-associated-diseases. Among the metabolites of ellagitannins that are produced in the gut microbiome, urolithin A (UA) has received an increasing attention as a novel candidate with anti-inflammatory and anti-oxidant effects. Here, we investigated the effect of UA on the suppression of pro-inflammatory molecules and NF-κB activation by targeting TLR4 signalling pathway. We also identified the influence of UA on Ca^2+^ entry, ROS production and DSBs availability in murine bone-marrow-derived macrophages challenged with lipopolysaccharides (LPS). We found that UA inhibits IκBα phosphorylation and supresses MAPK and PI3K activation. In addition, UA was able to reduce calcium entry, ROS production and DSBs availability. In conclusion, we suggest that urolithin A is a promising therapeutic agent for treating inflammatory diseases through suppression of NF-κB and preserving DNA through maintaining intracellular calcium and ROS homeostasis.

## Introduction

Antibiotic exposure in early life can lead to long-term alterations in the diversity, composition, and metagenomics content of the gut microbiota that may contribute to later onset of inflammatory bowel disease (IBD)^1,2^. IBD is a chronic inflammation of the gastrointestinal tract (GIT) which includes Crohn’s disease (CD) and ulcerative colitis (UC), and estimated to affect more than 0.4% of Europeans and North Americans^3^. It is well recognized that patients with IBD are at high risk for developing colorectal cancer (CRC)^4,5^. The nuclear factor-κB (NF-κB) signaling pathway plays a prominent role in the development and maintenance of most chronic diseases^6^. NF-kB is a key regulator and important player in linking inflammation to cancer development through its ability to upregulate various inflammatory molecules such as IL-1β, IL-6 and TNFα. It is a matchmaker between inflammation, IBD, cancer and diabetes^7,8^.

Intestinal inflammation is affected by the powerful anti-inflammatory effects of polyphenolic compounds^9^. Gut microbiota is able to convert dietary components such as polyphenols to a spectrum of metabolites^10,11^. Importantly, the health benefits rendered by consumption of several natural plant products (e.g. pomegranates, walnuts and berries) have been associated with high levels of polyphenolic compounds, specifically ellagitannins and ellagic acid^12,13^. Acid hydrolysis of ellagitannins releases free ellagic acid (EA)^14^ which are present in pomegranates, raspberries, strawberries, and walnuts^15^.

Ellagitannins-rich food has beneficial effects on IBD and other inflammatory diseases as arthritis and cancer^16^. The bioavailability of ellagitannins and ellagic acid is very low. They need further digestion by gut microbiota to produce bioactive molecules including urolithin compounds that can be easily absorbed^14,17,18^. In addition, only one in each three people has the microbiota that can perform this metabolism with maximum efficiency^19^. Short chain fatty acids (propionic acid, acetic acid and butyric acid) are one of the most popular metabolites of commensal microbes in the intestine which can contribute positively to the development of adaptive immunity T regulatory (Treg) cells^20^. Butyrate induces Treg cell differentiation in the gut mucosa, which reduces intestinal inflammation via secretion of anti-inflammatory cytokines such as IL-10^21,22^. Acetate has been recognised for its ability to reduce the autoimmune CD8^+^ T cells and thus inhibit inflammation in type 1 diabetes^23^.

Macrophages are one of the primary components of innate immune system, which plays a prominent role during the immune response to harmful stimuli such as bacteria and viruses^24^. Macrophages are widely spread throughout the tissues and produce various bioactive molecules which determine the final outcome of inflammation. Thus, therapeutic approaches targeting macrophages and their products can provide new opportunities for controlling inflammatory diseases^25^. Lipopolysaccharide (LPS) is the main component within the cell wall of Gram negative bacteria. It has been conventionally used to mimic the real infection triggering the release of inflammatory cytokines such as IL-1β, IL-6 or TNFα through activation of TLR4 signalling^26^.

The innate immune system provides the macrophages with pattern recognition system (PRRs) on their surface through which macrophages can sense pathogens and damaged cells through damage associated molecular patterns (DAMPs)^27^. The most important type of PRRs are toll-like receptors (TLRs). Studies have proposed that TLR2, TLR4, TLR5, TLR7, and TLR9 are the main PRRs, which stimulate inflammatory responses following the exposure of innate immune cells to pathogens or damaged cells^28–30^. These TLRs, through the stimulation of myeloid differentiation primary response 88 (MyD88), induce the upregulation of MAPK, NF-κB, and other transcription factors, leading to the secretion of pro-inflammatory cytokines, including IL-1, IL-6, IL-8, TNF, IL-33, and IFN-γ^30^. TLR2 and TLR4 have gained enormous importance due to their extreme ability of identifying distinct molecular patterns from invading pathogens. These PRRs not only act as innate sensors but also shape and bridge innate and adaptive immune responses. Furthermore, they play a crucial role in regulating the balance between Th1 and Th2 type of response essential for the survivability of the host^31^. Therefore, in this study, we attempted to analyze the ability of gut microbiota metabolites of ellagitannins urolithin A (UA) in reduction of inflammation induced by LPS (mimic the real scenario of bacterial infection) by targeting TLR4 signalling pathway (MAPK, NF-κB, and PI3K), pro-inflammatory cytokines, intracellular calcium, cellular ROS production and DNA double strand breaks (DSBs) in murine BMDMs.

## Results

### Urolithin A diminished the inflammatory miRNA expression in LPS-stimulated murine BMDMs

**Figure 1:**
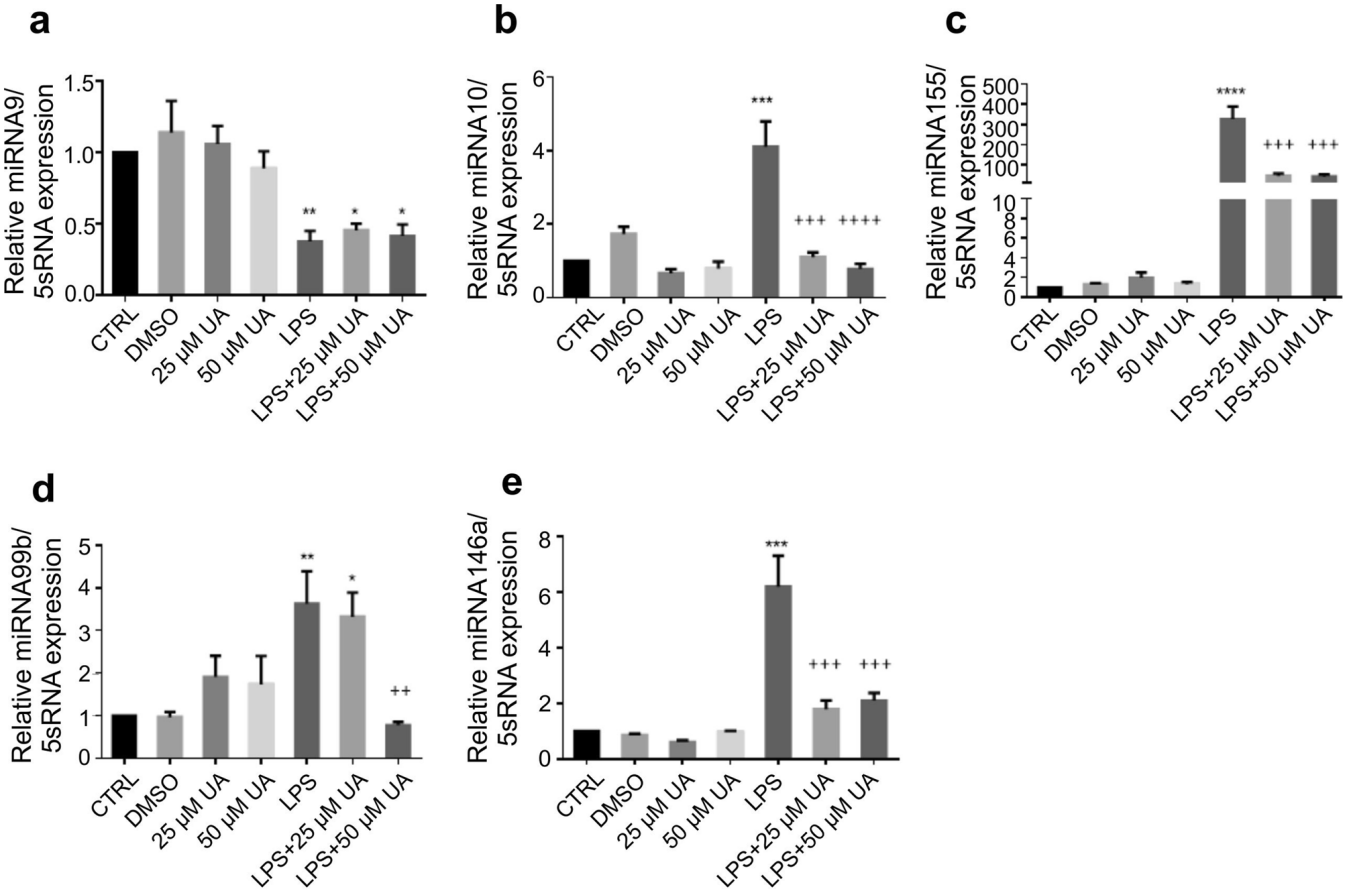
Urolithin A suppressed the inflammatory miRNA expression in LPS-stimulated murine BMDMs. Murine BMDMs were stimulated by 1 µg/ml of LPS in the presence or absence of UA (25 µM or 50 µM). The expression of miR-9 (**a**), miR-10 (**b**), miR-99b (**c**), miR.146a (**d**) and miR-155 (**e**) over 5S rRNA was evaluated in stimulated BMDMs after 72 h. The unstimulated and untreated BMDMs were used as control. BMDMs treated with DMSO were used as negative control. Arithmetic means ± SEM from seven independent experiments are depicted. One way ANOVA was used and *(*p* < 0.05), **(*P* 0.01), ***(*p* < 0.001), and ****(*p* < 0.0001) indicate statistically significant differences compared to control. ++(*P* 0.01), +++(*p* < 0.001), and ++++ (*p* < 0.0001) indicate statistically significant differences compared to LPS. Abbreviations: DMSO, dimethyl sulfoxide; UA, urolithin A; LPS, lipopolysaccharides; miR, micro RNA.

MicroRNAs (miRNA) are emerging as central regulators of inflammation. The post-transcriptional regulation is an important control mechanism for the expression of genes involved in inflammation like cytokines and chemokines^32,33^. Therefore, the influence of UA (25 µM or 50 µM) on inflammatory miRNA (Fig. 1) such as miR-9 (a), miR-10 (b), miR-99b (c), miR-146a (d), and miR-155 (e) in LPS-stimulated BMDMs were investigated. BMDMs receiving UA (25 µM or 50 µM) alone did not record any significant changes in examined miRNA expression except a slight non-significant elevation in miR-99b expression, whereas LPS-stimulation induced the expression of miR-10, miR-99b, miR-146a, and miR-155. The presented data demonstrate that treatment with UA (25 µM or 50 µM) induced a remarkable decrease in miR-10, miR-99b, miR-146a and miR-155 expression in LPS-stimulated BMDMs with values near to untreated control.

### Urolithin A blunted cellular ROS production in LPS-stimulated murine BMDMs

**Figure 2:**
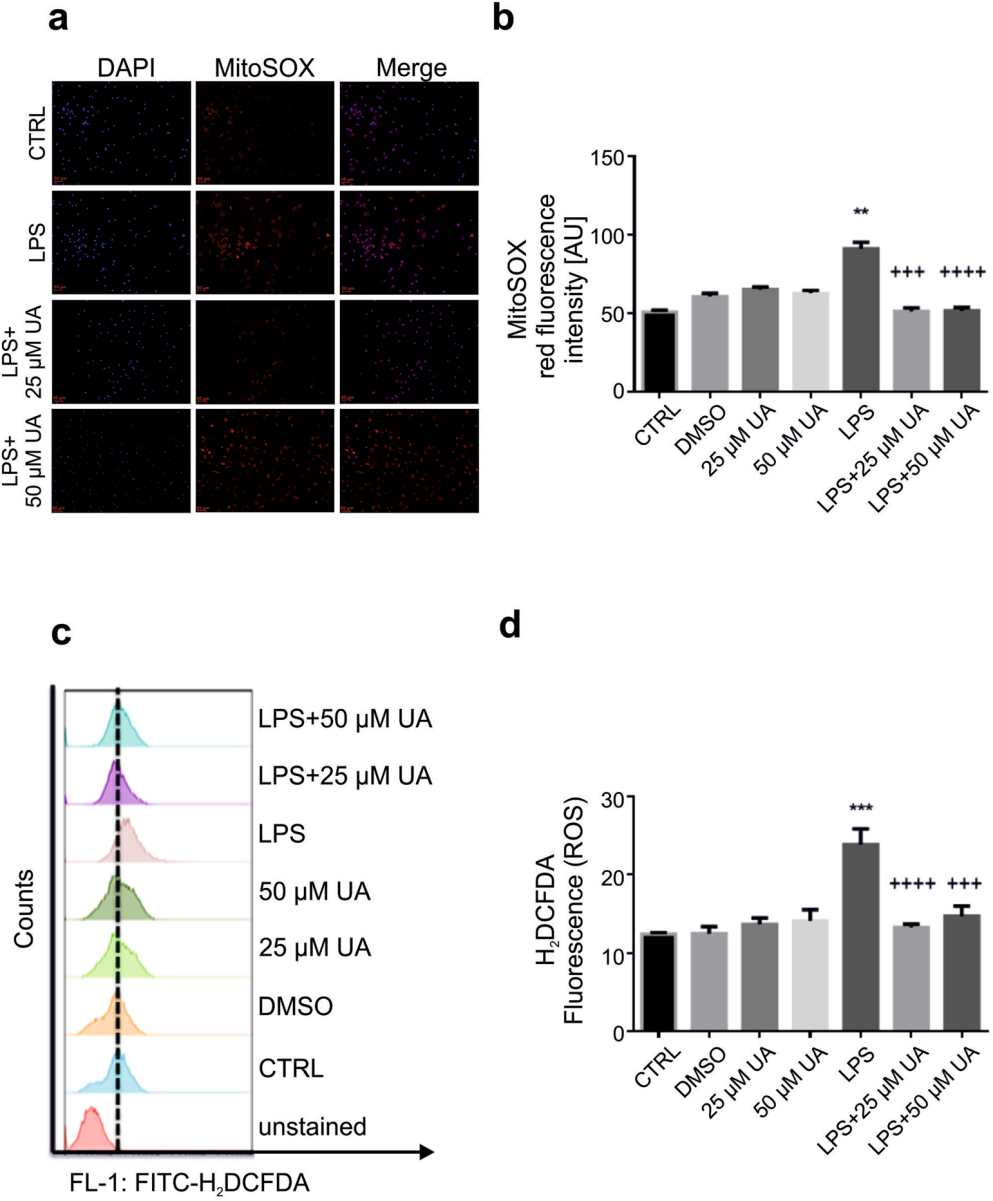
Urolithin A diminished cellular ROS production in LPS-stimulated murine BMDMs. (**a**) Murine BMDMs were stimulated by 1 µg/ml of LPS in presence or absence of UA (25 µM or 50 µM) for 48 h. LPS induced remarkable elevation of superoxide production (MitoSOX) demonstrated by red immunofluorescence which canceled with UA. Nuclei were counterstained with DAPI (blue). Images were taken with fixed exposure times for DAPI = 6 and MitoSOX = 750. The magnifications are 20-fold and scale bar represents 50 μm. (**b**) The graph represents a significant difference in mitochondrial ROS production compared to untreated control. (**c**) Representative original FACS histograms showing the effect of UA on ROS production (H_2_DCFDA) in LPS-stimulated murine BMDMs after 48 h. (**d**) The graph represents a significant difference in cellular ROS production compared to untreated control after 48 h. The unstimulated and untreated murine BMDMs were used as control. Murine BMDMs treated with DMSO were used as negative control. Arithmetic means ± SEM (n = 5–7). One way ANOVA was used and **(*P* < 0.001) and ***(*p* < 0.001) indicate statistically significant differences compared to control, whereas +++(*p* < 0.001), and ++++(*p* < 0.0001) indicate statistically significant differences compared to LPS. Abbreviations: DMSO, dimethyl sulfoxide; UA, urolithin A; LPS, lipopolysaccharides.

Dysregulated ROS production can enhance tumor formation via the activation of various oncogenic signalling pathways, DNA mutation, immune escape, tumor microenvironment and metastasis^34^. Thus, the level of ROS production was evaluated using fluorescence microscopy and flow cytometry in LPS-stimulated murine BMDMs with or without UA (25 µM or 50 µM). Murine BMDMs were labeled with MitoSOX™ Red reagent, which can be detected in red fluorescence when oxidized by superoxide, and nuclei were stained with blue-fluorescent DAPI (Fig. 2). The obtained results show that neither UA nor DMSO alone induced any significant changes on superoxide production in murine BMDMs after 48 h (Supplementary Fig. 1). Data represented in Fig. 2a demonstrate that after 48 h of LPS stimulation, BMDMs exhibit red immunofluorescence, indicating a highly significant increase in superoxide production. UA (25 μM or 50 μM) was able to inhibit the superoxide production (Fig. 2a, b).

In parallel, we studied the effect of UA (25 µM or 50 µM) on ROS production using H2DCFDA dye after 48 h (Fig. 2c, d). The obtained results indicate that neither DMSO nor UA (25 µM or 50 µM) alone induced any significant changes in ROS production. UA was, however, able to abolish the remarkable elevation of ROS production induced by LPS after 48 h (Fig. 2c, d).

### Urolithin A reduced intracellular Ca^2+^ concentration in LPS-stimulated murine BMDMs

As the interactions between ROS and calcium signaling can be considered as bidirectional, wherein ROS can regulate cellular calcium signaling and calcium signaling is essential for ROS production^35^. Thus, the influence of UA (25 µM or 50 µM) on calcium entry in LPS-stimulated BMDMs after 24 h and 48 h was evaluated. The calcium influx or altered intracellular calcium concentration can be measured using a cell permeant dye, Fluo-4, which becomes fluorescent upon binding to calcium.

**Figure 3:**
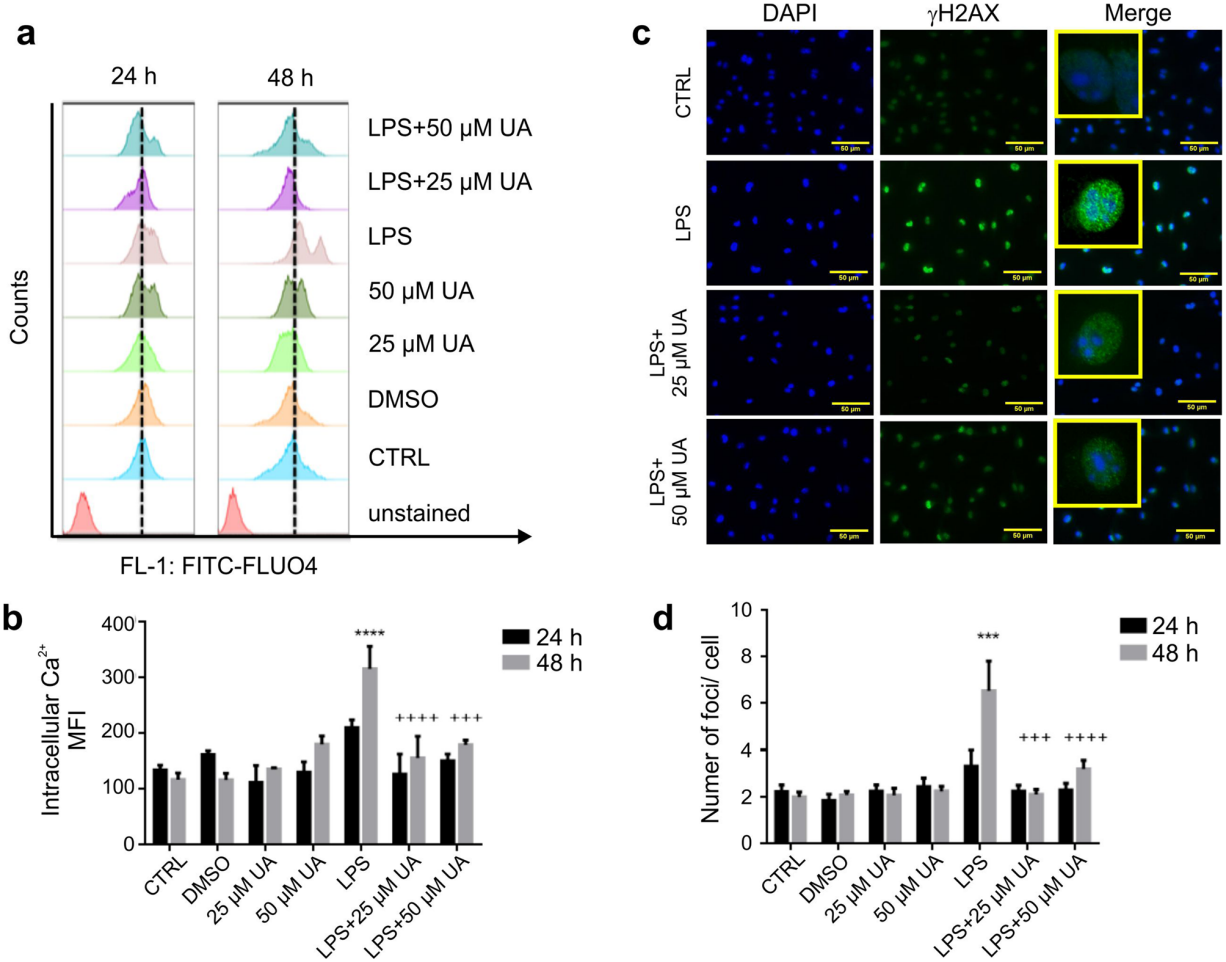
Urolithin A minimized the intracellular calcium concentration and ameliorated DSBs in LPS-stimulated murine BMDMs. (**a**, **b**) Murine BMDMs were stimulated with 1 µg/ml LPS in the presence or absence of UA (25 µM or 50 µM) for 24 h or 48 h. (**a**) Representative FACS histograms showing the effect of UA on stimulated BMDMs after 24 h and 48 h. The reference line was set at the peak of the control. (**b**) Arithmetic means ± SEM (n = 5–7) show a significant difference in intracellular Ca^2+^ concentration between control and treated groups after 24 h and 48 h. The unstimulated BMDMs were used as untreated control. DMSO was used as negative control. The intracellular Ca^2+^ was measured with flow cytometry. (c) Murine BMDMs were stimulated by 1 µg/ml of LPS with or without UA (25 µM or 50 µM) for 2 h (images not shown) and 48 h. After 48 h, LPS induced DSBs indicated by a prominent green immunofluorescence for Ser139-phosphorylated H2AX. Treatment with UA recorded a remarkable decrease in number of γH2AX foci. Nuclei were counterstained with DAPI (blue). The magnifications are 20-fold and scale bar represents 50 μm. The unstimulated and untreated BMDMs were used as control. DMSO was used as negative control. (**d**) Graph indicates the number of γH2AX foci per cell after indicated time points. Representative images and arithmetic means ± SEM from four independent experiments (600 cells were counted). Two way ANOVA was used and ***(*p* < 0.001) indicate statistically significant differences compared to respective control, whereas +++(*p* < 0.001), and ++++(*p* < 0.0001) indicate statistically significant differences compared to LPS. Abbreviations: DMSO, dimethyl sulfoxide; UA, urolithin A; LPS, lipopolysaccharides.

Results represented in Fig. 3 reveal that UA (25 μM or 50 μM) was able to revoke the upregulation of intracellular calcium induced by LPS in BMDMs after 48 h. While, after 24 h a non-significant increase was recorded (Fig. 3b). In addition, the obtained results show that neither DMSO nor UA (25 µM or 50 µM) alone induced any significant change during the time intervals.

### Urolithin A induced prominent decrease in DNA double strand breaks (DSBs) in LPS-stimulated murine BMDMs

Oxidative stress induced by extracellular and intracellular production of ROS is a fundamental mechanism that contributes to DNA damage. Overproduction of ROS that exceeds defense mechanisms can damage intracellular macromolecules, including nucleic acids, with the formation of DNA adducts and DNA strand breaks that may result in mutations^36,37^. As LPS induced upregulation of calcium entry and ROS production in BMDMs, we were interested in studying and evaluating the impact of LPS-stimulation on DSBs in the presence or absence of UA in BMDMs.

Murine BMDMs were fixed and immunostained for phosphorylated H2AX (Ser139) at 2 h (images are not shown) and 48 h (Fig. 3c) after LPS exposure. A higher significant number of γH2AX foci were only observed after 48 h (Fig. 3d) of LPS stimulation and UA was able to induce a prominent decrease in DSBs achieving values near to untreated control (Fig. 3d). In addition, neither UA (25 µM or 50 µM) nor DMSO alone induced any significant effect on DSBs in BMDMs (Supplementary Fig. 2).

### Urolithin A downregulated the inflammatory cytokines production and mRNA expression in LPS-stimulated BMDMs

**Figure 4:**
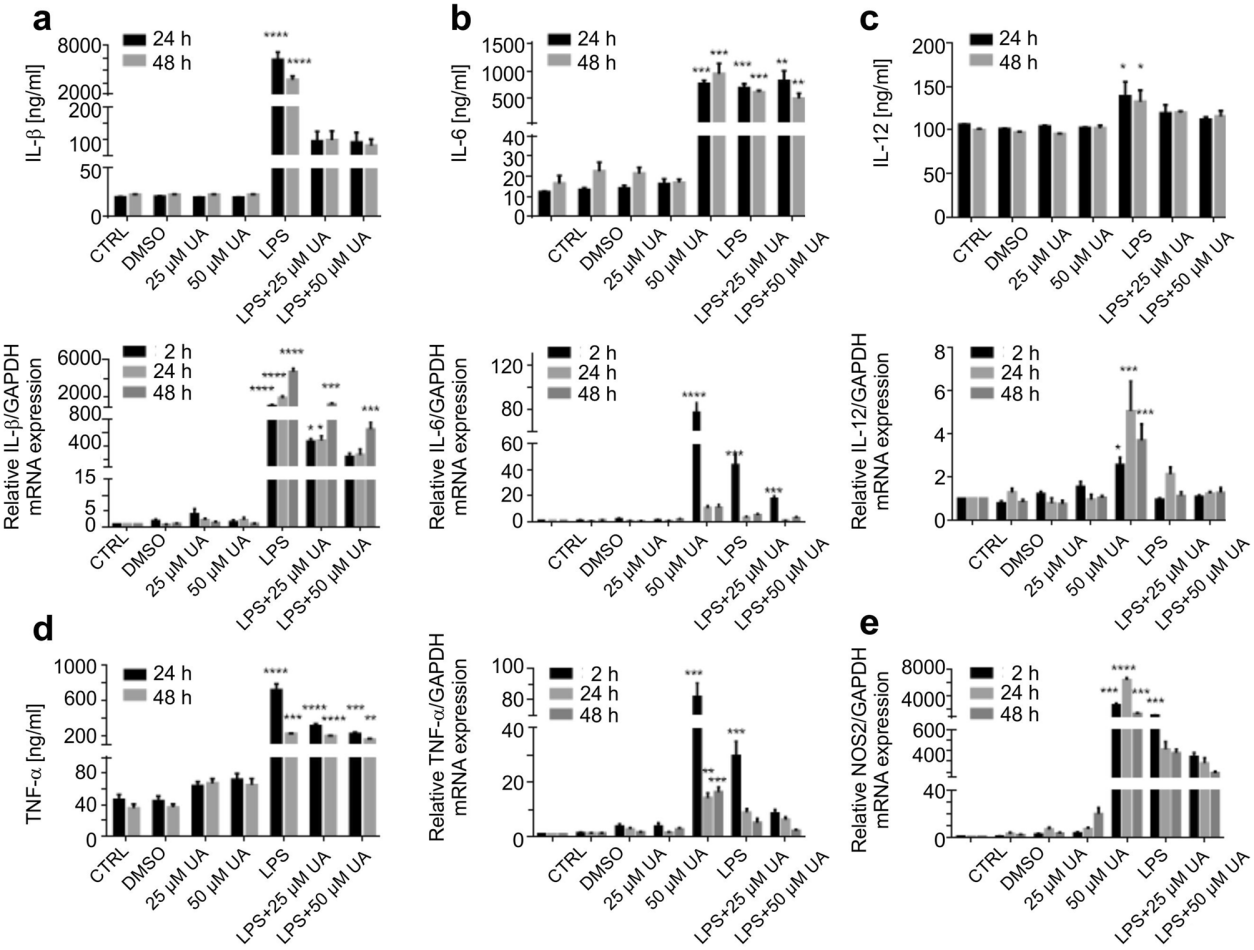
Urolithin A reduced the pro-inflammatory cytokine production and mRNA expression in LPS-stimulated murine BMDMs. Murine BMDMs were stimulated by 1 µg/ml of LPS in the presence or absence of UA (25 µM or 50 µM). Subsequently, expression of the pro-inflammatory cytokines IL-1β (**a**), IL-6 (**b**), IL-12 (**c**), TNF-α (**d**), and NOS2 (**e**) were measured in BMDMs by qRT-PCR (over GAPDH) and ELISA at depicted time points. The unstimulated and untreated BMDMs were used as control. BMDMs treated with DMSO were used as negative control. Arithmetic means ± SEM from seven independent experiments are depicted. Two way ANOVA was used and *(*p* < 0.05), **(*P* 0.01), ***(*p* < 0.001), and ****(*p* < 0.0001) indicate statistically significant differences compared to respective control. Abbreviations: DMSO, dimethyl sulfoxide; UA, urolithin A; LPS, lipopolysaccharides; IL, Interleukin; GAPDH, glyceraldehyde 3-phosphate dehydrogenase; and TNF, tumor necrosis factor; NOS, nitric oxide synthase.

IL-1β, IL-2, IL-6, IL-12 and TNFα (Fig. 4) are some of the pro-inflammatory cytokines that participate in the prolonging of chronic inflammation. These cellular messengers should be suppressed to avoid further inflammatory processes.

In addition, the anti-inflammatory IL-4, IL-10, IFNγ, TGFβ (Supplementary Fig. 3) as well as NOS2 (Fig. 4) were also evaluated either by ELISA and/or qRT-PCR in LPS-stimulated murine BMDMs. LPS is known to induce a typical M1 phenotype^38^. The presented data in Fig. 4 reveals that treatment with UA (25 µM or 50 µM) was able to induce significant depressions in IL-1β (a), IL-6 (b), IL-12 (c), TNF-α (d), and NOS2 (e) expression with values near to untreated control. On the other hand, the data presented in Supplementary Fig. 3 revealed that UA induced a remarkable decrease in IFN-γ (a), TGF-β (b), IL-10 (c), and IL-2 (d) expression, but a remarkable increase in IL-4 (e) expression was recorded in the presence of UA to LPS-stimulated BMDMs (Supplementary Fig. 3).

The notable increase in IL-10 expression was registered after 2 h and down regulated at later time points in the presence or absence of LPS and/or UA. Notably, LPS induced a significant elevation in the mRNA expression of IFN-γ which did not translated into protein during the depicted time points as compared to their respective control groups. The administration of UA (25 µM or 50 µM) alone induced a notable dose dependent increase in IL-1β, IL-2, IL-4, IL-6, NOS2, TGF-β and IFN-γ mRNA expression.

### Urolithin A reduced TLR4 expression in LPS-stimulated murine BMDMs

The presented data in Supplementary Fig. 4 reveals that the stimulation of BMDMs with 1 µg of LPS induced significant elevation in TLR4 (72 h) expression. Administration of UA to LPS-stimulated BMDMs induced remarkable decreases in TLR4 expression (Supplementary Fig. 4). Murine BMDMs receiving both concentrations of UA (25 µM or 50 µM) alone had no significant effect on TLR4 expression.

### Urolithin A revoked IκBα phosphorylation in LPS-stimulated murine BMDMs

**Figure 5:**
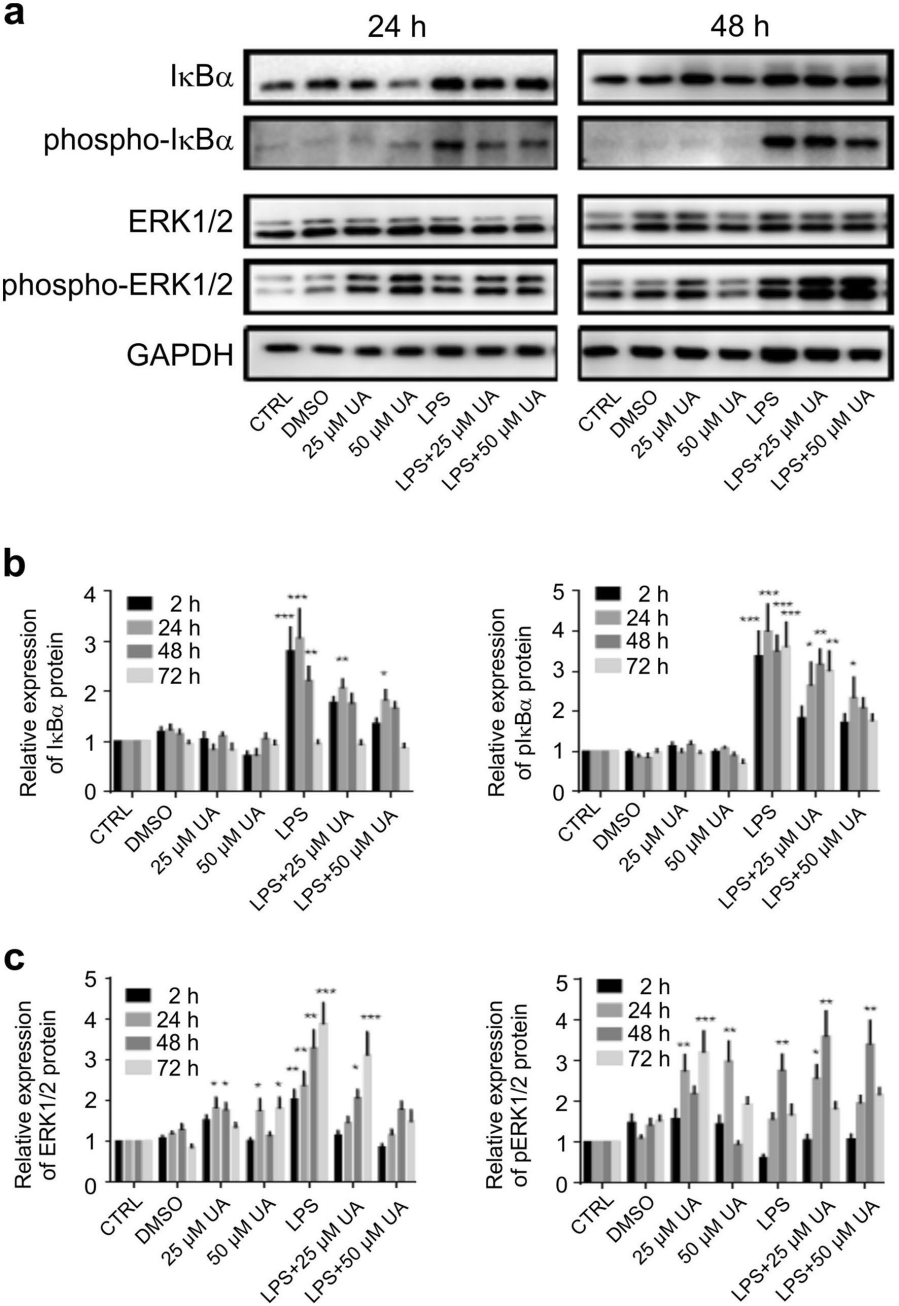
Effect of urolithin A on IκBα and MAP kinase ERK1/2 expression and phosphorylation in LPS-stimulated murine BMDMs. Murine BMDMs were stimulated by 1 µg/ml of LPS in the presence or absence of UA (25 µM or 50 µM) and harvested at indicated time intervals, followed by western blot analysis. Phosphorylation of IκBα (**a**, **b**) and MAP kinases ERK1/2 (**a**, **c**) were monitored by immunoblot using pIκBα (Ser32/36) monoclonal antibodies and phospho-p44/42 MAP kinase (Thr202/Tyr204) polyclonal antibodies, respectively at depicted time points. Subsequently, blots were stripped and re-incubated with antibodies against total IκBα and ERK1/2. GAPDH served as a loading control. The unstimulated and untreated BMDMs were used as control. BMDMs treated with DMSO were used as negative control. Representative images and arithmetic means ± SEM from five independent experiments are depicted. Two way ANOVA was used and *(*p* < 0.05), **(*P* 0.01), and ***(*p* < 0.001) indicate statistically significant differences compared to respective control. Full length blots are found in the Supplementary File 9. Abbreviations: DMSO, dimethyl sulfoxide; LPS, lipopolysaccharides; UA, urolithin A; GAPDH, glyceraldehyde-3-phosphate dehydrogenase.

NF-κB is the master transcription factor in inflammatory processes and is normally inhibited by the protein IκBα. BMDMs stimulated with 1 µg of LPS in the presence or absence of UA were evaluated for the level of phospho-IκBα by immunoblot analysis. Data presented in Fig. 5 show that UA (25 μM or 50 μM) was able to impair the upregulation of total IκBα and pIκBα (Fig. 5a, b) induced by LPS-stimulation, achieving a dose dependent decrease in pIκBα compared to those receiving LPS alone during the time intervals. A representative image of protein expression at 2 h and 72 h is shown in Supplementary Fig. 5.

### Urolithin A blunted MAPK activation in LPS-stimulated murine BMDMs

**Figure 6:**
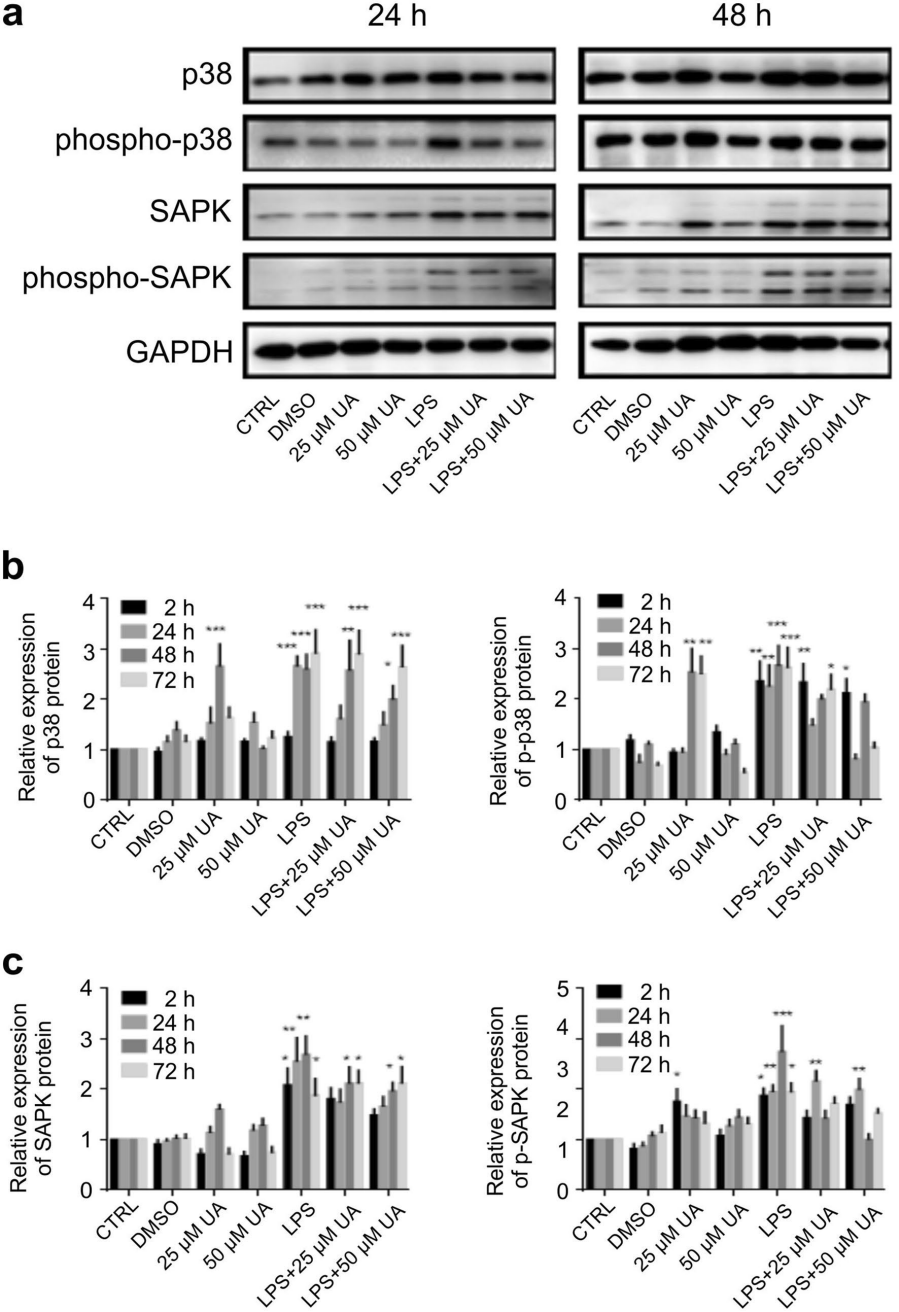
Effect of urolithin A on MAP kinase p38 and SAPK/JNK expression and phosphorylation in LPS-stimulated murine BMDMs. Murine BMDMs were stimulated by 1 µg/ml of LPS in the presence or absence of UA (25 µM or 50 µM) and harvested at indicated time intervals, followed by western blot analysis. Phosphorylation of MAP kinase p38 (**a**, **b**), and SAPK/JNK (**a**, **c**) were monitored by immunoblot using phospho-p38 MAP kinase (Thr180/Tyr182) and phospho-SAPK/JNK MAP kinase (Thr183/Tyr185) monoclonal antibodies, respectively at depicted time points. Subsequently, blots were stripped and re-incubated with antibodies against total p38 and SAPK/JNK. GAPDH served as a loading control. The unstimulated and untreated BMDMs were used as control. BMDMs treated with DMSO were used as negative control. Representative images and arithmetic means ± SEM from five independent experiments are depicted. Two way ANOVA was used and *(*p* < 0.05), **(*P* 0.01), and ***(*p* < 0.001) indicate statistically significant differences compared to respective control. Full length blots are found in the Supplementary File 9. Abbreviations: DMSO, dimethyl sulfoxide; LPS, lipopolysaccharides; UA, urolithin A; GAPDH, glyceraldehyde-3- phosphate dehydrogenase.

The effect of UA on MAPK involved in the regulation of TLR4 expression was investigated after LPS-stimulation in murine BMDMs. Total and phosphorylated ERK1/2 (Fig. 5a, c), p38 (Fig. 6a, b), and SAPK/JNK (Fig. 6a, c) were analysed by immunoblot and normalized to GAPDH at the depicted time intervals. UA alone did not significantly modify total p38 and SAPK/JNK expression, but significantly upregulated total ERK1/2. The administration of UA (25 μM or 50 μM) to LPS-stimulated BMDMs induced a dose dependent decrease in p38, and SAPK/JNK phosphorylation and had no effect on ERK1/2 phosphorylation (Fig. 5). A representative image of protein expression at 2 h and 72 h is shown in Supplementary Fig. 5.

### Urolithin A suppressed AKT and mTOR stimulation induced by LPS-stimulation in murine BMDMs

**Figure 7:**
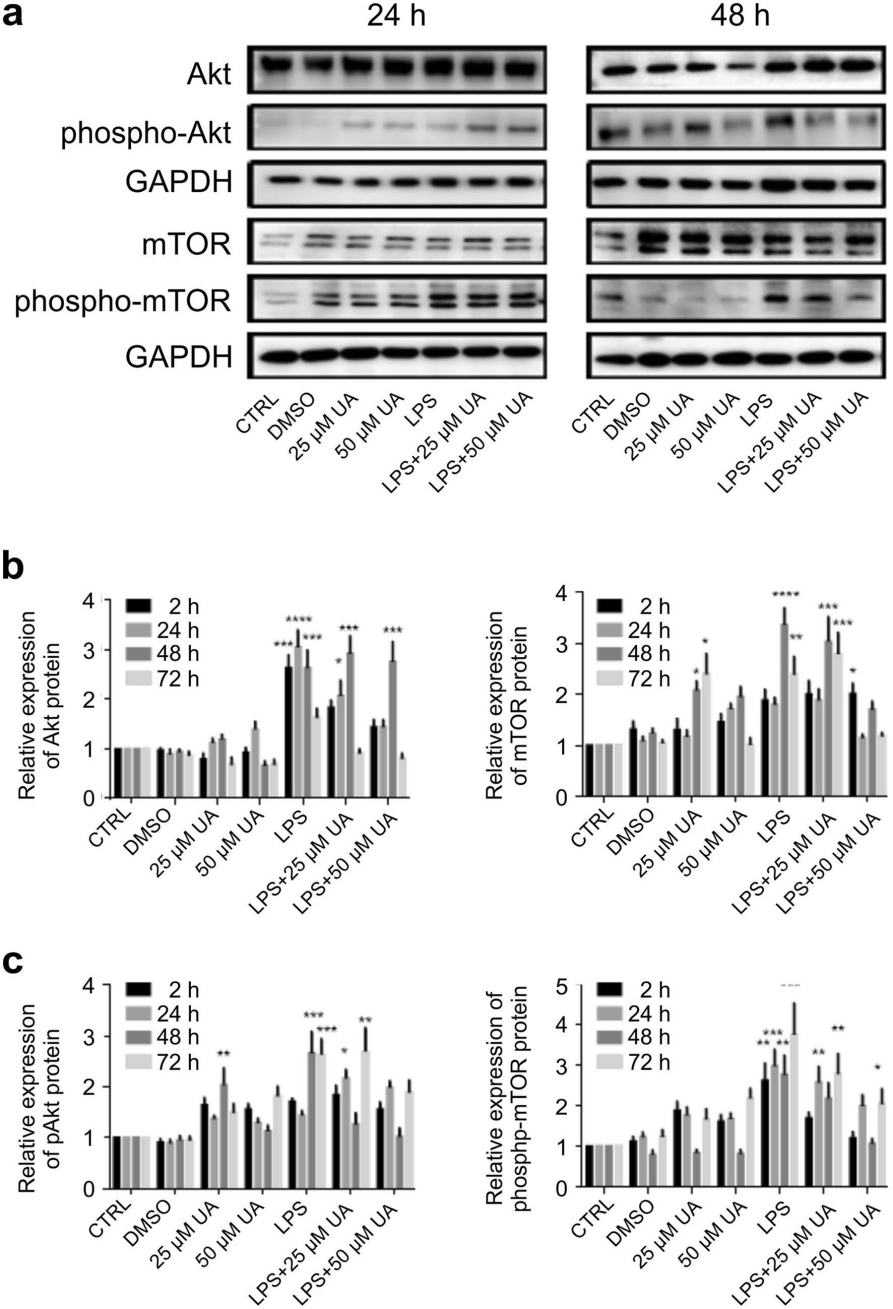
Urolithin A diminished PI3K/AKT/mTOR activation in LPS-stimulated murine BMDMs. Murine BMDMs were stimulated by 1 µg/ml of LPS in the presence or absence of UA (25 µM or 50 µM) and harvested at indicated time intervals followed by western blot analysis. Phosphorylation of AKT (**a**, **b**) and mTOR (**a**, **c**) were monitored by immunoblot using pAKT (Ser473) and phospho-mTOR (Ser2448) monoclonal antibodies. Subsequently, blots were stripped and re-incubated with antibody against total AKT and mTOR. GAPDH served as a loading control. The unstimulated and untreated BMDMs were used as control. BMDMs treated with DMSO were used as negative control. Representative images and arithmetic means ± SEM from five independent experiments are depicted. Two way ANOVA was used and *(*p* < 0.05), **(*P* 0.01), ***(*p* < 0.001), and ****(*p* < 0.0001) indicate statistically significant differences compared to respective control. Full length blots are found in the Supplementary File 9. Abbreviations: DMSO, dimethyl sulfoxide; LPS, lipopolysaccharides; UA, urolithin A; GAPDH, glyceraldehyde-3- phosphate dehydrogenase.

Several TLRs induce the PI3K/Akt pathway which can regulate the immune response in a negative or positive manner^39^. Both Akt and mTOR are principal signalling pathways that orchestrate the response of macrophages to various metabolic and inflammatory signals^40^. In order to clarify the mechanisms of TLR4 regulation by LPS and how the gut microbiota metabolite urolithin A can drive these signalling pathways, expression of PI3K/AKT/mTOR was evaluated. As shown in Fig. 7, UA (25 µM or 50 µM) alone did not significantly modify total AKT expression but significantly increased total mTOR expression. LPS significantly increased both AKT (Fig. 7a, b) and mTOR (Fig. 7a, c) phosphorylation within 2 h and continued up to 72 h (Supplementary Fig. 5), an effect dose dependently blunted by UA.

## Discussion

**Figure 8:**
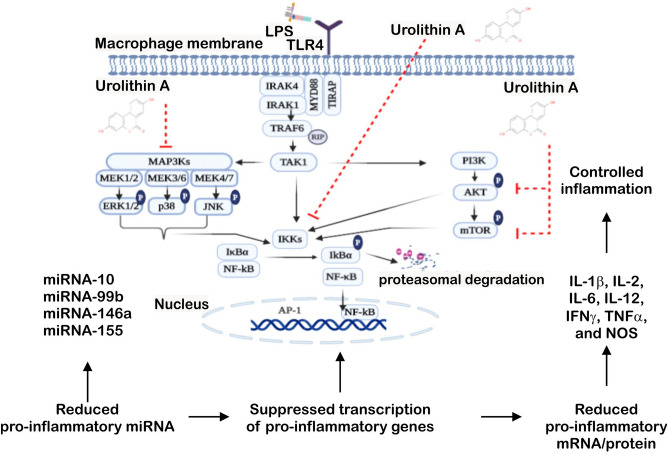
Urolithin A controlled NF-kB inflammatory response. LPS stimulates TLR4 signalling cascades. Urolithin A impairs the NF-kB activation through the inactivation of IKKs. Urolithin A suppresses MAPKs activation through blocking ERK, p38 and JNK phosphorylation. Additionally, urolithin A inhibits the phosphorylation of AKT and mTOR protein. Abbreviation: LPS; lipopolysaccharide; IR, ionizing radiation; TIRAP, TIR adaptor protein; MyD88, myeloid differentiation primary-response gene 88; IRAK, interleukin-1 receptor associated kinase; TRAF, TNF receptor associated factor; RIP, receptor-interacting protein kinases; TAK, transforming growth factor beta-activated kinase 1; IKK, IκB kinase; MAPK, mitogen activated protein kinase; PI3K, phosphatidylinositol 3-kinases; mTOR, mechanistic target of rapamycin; ERK, extracellular regulated kinase; JNK, c-Jun N-terminal kinase; NF-κB, nuclear factor-κB; IκB, kinase complex; AP, activator protein; IL, interleukin; IFN, interferon; TNF, tumor necrosis factor; and NOS, nitric oxide.

The obtained results reveal a powerful anti-inflammatory effect of the gut microbiota UA, which is paralleled by suppression of MAPK and PI3K activation and impairment of IkBα phosphorylation as well as quenched production of pro-inflammatory miRNA, cytokines and mediators in LPS-stimulated BMDMs (Fig. 8). In addition, the anti-oxidant ability of UA manifested by its ability to preserve DNA breaks through abolishing the cellular ROS production and suppressing calcium entry in LPS-stimulated BMDMs.

TLRs play essential roles in triggering innate immune responses against bacteria and viruses^41,42^. TLR4, which recognizes LPS, is highly expressed by macrophages, dendritic cells, and monocytes^43^. Activation of TLR4-dependent signalling processes in macrophages was estimated with different methods, especially IκBα degradation and NF-κB phosphorylation and nuclear translocation followed by a series of intracellular signaling pathways, which finally leads to the transcription/translation of inflammatory cytokines^44^. Our data revealed that the treatment with UA induced remarkable changes on LPS-stimulated BMDMs. In order to elucidate the effect of UA on TLR4 expression and the underlying molecular mechanism by which UA exerts anti-inflammatory activity, its influence on the activation of the NF-κB, MAPK and PI3K pathways were evaluated in BMDMs challenged with LPS. Once LPS ligate to TLR4, a series of intracellular signaling pathways are activated, which ultimately lead to the transcription/translation of inflammatory molecules^45,46^. As shown in Fig. 5, UA inhibits the phosphorylation of IκBα and prevents the nuclear translocation of NF-κB. Such impairment of NF-κB activation may be due to the suppressive effect of UA against IL-1β, IL-2, IL-6, IL-12, TNFα and NOS2 (Fig. 4). In addition, both MAPK (Figs. 5, 6) and PI3K (Fig. 7) pathways are also stimulated by LPS and mediate the transcription of various miRNA, cytokines and chemokines^47,48^. Our results revealed that UA suppressed the phosphorylation of MAPK (p38 and SAPK/JNK) (Figs. 5, 6), AKT and mTOR (Fig. 7). These results suggest that these two signaling pathways may be possible targets of UA and could mediate the reduction of NOS2, IL-1β, IL-2, IL-6, IL-12 and TNFα levels.

The consumption of several natural plant products such as pomegranates, walnuts and berries results in the production of high levels of polyphenolic compounds, specifically ellagitannins and ellagic acid^12,13^. Acid hydrolysis of ellagitannins releases free ellagic acid (EA)^14^. The absorption of ellagitannins and ellagic acid is very low and so, the unabsorbed compounds are further metabolised by gut microbiota to bioactive molecules including the different urolithin compounds A, B, C, and D^14,17^. Among urolithins, urolithin A (UA; 3,8-dihydroxybenzo[c]chromen-6-one) is the most relevant urolithin and has been shown to influence the microbiota composition in rat models^49^, but the significance of these changes remain to be established. The gut bacterial metabolites such as SCFAs including propionic acid, acetic acid, and butyric acid can positively contribute to activation of the adaptive immunity^20,23,50^.

UA has an anti-inflammatory property in inflammatory bowel disease and improves the gut permeability^51^. It has also been observed that UA may play an important role in the inhibition of certain cancers, such as colorectal or prostate cancers^52,53^. In addition to its anti-inflammatory activity, UA preserves gut barrier integrity by increasing tight junction proteins induced by activation of aryl hydrocarbon receptor through the NF-E2 p45-related factor 2 (NRF2)-dependent pathway^49,54^.

MicroRNAs are involved in all facets of immune system development starting from hematopoiesis to activation during the immune response to harmful stimuli^55^. Dysregulation of miRNA can induce immune disturbance leading to various diseases including cancer^56^. LPS is a potent stimulator of several miRNA including miR-146a, miR-132 and miR-155^57^.

Promoter analysis studies recognized miR-146a as NF-κB-inducible miRNA. Also, the study identified a crucial role of miR-146 in controlling TLR and cytokine signalling via a negative feedback regulation loop^57^. Our results show that UA was able to supress the expression level of miR-10, miR-99b, miR146a and miR-155 after LPS-stimulation. On the contrary, Bazzoni, Rossato et al.^58^ reported that miR-9 is activated by TLR4 in human PMNs and monocytes targeting NF-κB subunit p50. Therefore, suppressing the production of a pro-inflammatory cytokine as inhibition of NF-κB and MAPK activation may influence or mediate the reduction of miRNA expression.

Functional properties of the immune cells, including activation, chemokine and cytokine expression are further regulated by Ca^2+^ signalling^59,60^. Our data revealed that UA abolished the upregulation of intracellular calcium levels induced by LPS stimulation. Such inhibition may be achieved through the modulation of IP3R activity where UA was able to decrease the AKT phosphorylation. In parallel, UA quenched the elevation in mitochondrial and cellular ROS production induced by LPS stimulation. UA treatment apparently blocked ROS generation and restored the balance of the intracellular redox state by attenuating oxidative stress. In agreement with previous reports^61,62^, our results show that excessive ROS production induced by LPS stimulates MAPK and NF-κB pathways and initiates inflammatory responses. In addition, our data revealed that excessive ROS production and calcium entry induced by LPS stimulate PI3K/AKT/mTOR pathways. Such decrease in ROS production and entry of calcium was concomitant with prominent reduction in DNA double strand breaks induced by LPS stimulation after 48 h. This result was in agreement with the findings of Chumduri, Gurumurthy et al., who indicated that LPS can induce DSBs either directly or indirectly through reactive oxygen species^63^. In addition, Qiao, Huang et al. revealed that LPS‐induced DSBs activated the NF‐κB signalling pathway in human dental pulp tissues^64^.

Taken together, the results presented in this study demonstrate that UA differentially modulated the production of inflammatory miRNA and cytokines and suppressed LPS-induced effects on NOS2, ROS production, intracellular calcium, and DNA double strand breaks. These effects are most likely mediated by the suppression of IκBα phosphorylation, MAPK (p38 and JNK) and PI3K/AKT/mTOR signaling pathways (Fig. 8). Our findings highlight the potential of UA as a novel therapeutic agent for treating inflammatory diseases such as IBD. Further in vivo studies are essential to confirm the therapeutic or preventive effect of UA in animal disease models.

## Materials and methods

### Mice

C57BL/6 J mice between 8–12 weeks of age (both male and female) were used for experiments. All experiments were performed according to the EU Animals Scientific Procedures Act and the German law for the welfare of animals. All procedures were approved by the authorities of the state of Baden-Württemberg, i.e. Regierungspräsidium Tübingen.

### Bone marrow cells isolation, culture and treatment

Bone marrow cells (BMCs) isolated from femurs and tibias under sterile conditions were cultured in DMEM media (#61965-026- Life Technologies, Germany), supplemented with 10% FBS (#10270-106 - Life Technologies, Germany), 100 U/ml penicillin/streptomycin (#P4333- Sigma-Aldrich, Germany), 10% MEM Non-essential Amino Acid Solution (#M7145- Sigma-Aldrich, Germany), 0.1% 2-Mercaptoethanol (#M7522- Sigma-Aldrich, Germany) and 20% (v/v) L929 cell-condition medium (LCM) as a source of M-CSF. After 7 days in culture, adherent bone marrow–derived macrophages (BMDMs) were more than 90% pure. The efficiency of the differentiation is assessed using FACS analysis surface antigen expression. BMDMs were stimulated with 1 µg/ml LPS (*Escherichia coli* O111:B4, #L4391, Sigma-Aldrich, Germany) in the presence or absence of (25 µM or 50 µM) urolithin A (UA, #1143-70-0, Santa Cruz, Germany) for 2 h, 24 h, 48 h and 72 h. The untreated and unstimulated BMDMs were used as control and those received Dimethyl sulfoxide (DMSO - #A994-2, Roth, Germany) only were used as negative control.

### Phenotypic characterization of BMMs with directly conjugated cell surface antibodies

BMMs were detached by Accutase (#SCR005, Sigma-Aldrich) and collected in a 96 well plate, washed with 1 × PBS (#D8537, Sigma Aldrich, Germany), then centrifuged and re-suspended in 50 μl of fresh 1 × PBS. After that, 0.5 μl fluorescently-labelled-antibodies CD11b-PE (#12-0112-82), F4/80-FITC (#11-4801-81) and MHCII–APC (#17-5321-82) were added to each well. Cells were incubated in the dark for 30–45 min at 4 °C. After incubation, BMDMs were washed and acquired on the flow cytometry- BD FACS Calibur™ (BD Bioscience, Germany). The harvested BMCs (20.000 cells) were relatively favored to differentiate into macrophages (Supplementary Fig. 6) with CD11b^high^ (almost 100%), F4/80^high^ (90% ± 7%) and MHCII^low^ (10% ± 4%). In addition, the second characterization after LPS-stimulation in presence or absence of UA revealed that all groups are still F4/80^high^, CD11b^high^ and MHCII^low^ (Supplementary Fig. 7). The data were analysed by *FlowJo* software (FLOWJO, LLC, USA).

### Influence of urolithin A on the viability of murine BMDMs

BMDM (20.000 cells) were isolated, stimulated and treated as described above. The percentage of apoptotic cells was estimated by flow cytometry using the AnnexinV apoptosis detection kit FITC (#88-8005- 72, eBioscience) in accordance with the manufacturer’s instructions. Briefly, BMDMs were collected, washed and re-suspended in 1 × binding buffer containing Annexin V-FITC solution (1:50 dilution). After that, BMDMs (20.000 cells) were incubated at room temperature for 15 min, protected from light, and washed again. After adding Propidium Iodide solution (1:100 dilution), BMDMs were incubated at room temperature in the dark for 10 min prior to flow cytometry for cell apoptosis analysis^65,66^. The observed data show that more than 95% of the untreated control group were viable and non-apoptotic (Annexin V^−^ PI^−^). Dot plots show the effects of UA on LPS-stimulated BMDMs after 72 h (Supplementary Fig. 8a, c). BMDMs were treated with varying concentration of UA (for 72 h) ranging from 10–300 µM to demonstrate the dose dependent effect on apoptosis (Supplementary Fig. 8B). The apoptotic effects start to appear at 100 µM and according to these results, 25 µM UA and 50 µM UA were used for further experiments. Data were analyzed by *Flowjo* software (FLOWJO, LLC, USA).

### Measurements of intracellular calcium and reactive oxygen species (ROS) production

BMDMs were treated as desired and then collected and washed once and re-suspended in 96 well plates with 200 μl of 1 × PBS. In the following, 1 μM Fluo-4 (#F14200, Invitrogen, Germany) or 10 nM of ROS sensitive dye 2′,7′-Dichlorofluorescin diacetate (H2DCFDA, #D6883, Sigma-Aldrich, Germany) were added. BMDMs were incubated for 30 min at 37 °C in the dark and then washed twice with 1 × PBS buffer. Finally, BMDMs were re-suspended in 200 μl of PBS prior to flow cytometry (FL1 channel) for measurement with a FACSCalibur™. 20,000 cells/sample in the flow cytometer were acquired using the recommended software for data acquisition. Obtained values were corrected for autofluorescene of control cells without dye^66,67^. Geometric mean of the FL-1 signal intensity was used to show the amount of Fluo-4 and H2DCFDA fluorescence intensity (separate measurements). Data were analyzed by *FlowJo* software (FLOWJO, LLC, USA).

### MitoSOX Red mitochondrial superoxide indicator

BMDMs (10.000 cells per well) were grown, stimulated as indicated in X-well tissue culture chamber (#94.6150.401, Sarstedt, Germany), and further incubated for 48 h. BMDMs were washed with PBS and subsequently incubated for 10 min at 37 °C in 500 µl of 5 µM MitoSOX red reagent (#M36008D, Thermo Fisher Scientific, USA) protected from light. BMDMs were washed three times and one drop of ProLong GOLD Anti-fade Mountant with DAPI (#P36931, Thermo Fisher Scientific, USA) was added per well. The fluorescence imaging was performed with an Axiophot Zeiss microscope using a digital camera with *AxioVision 4.8* software. The obtained images were analysed by *Fiji ImageJ* software.

### γH2AX for DNA double strand breaks

BMDMs (10.000 cells per well) were seeded on a X-well tissue culture chamber, treated as indicated and further incubated for 2 h and 48 h. BMDMs were washed with 500 µl of PBS per well (using orbital mixer for 5 min) then fixed with 4% (v/v) formaldehyde (#158127, Sigma-Aldrich, Germany) for 10 min at RT, permeabilized for 10 min at RT with 0.1% (v/v) Triton-X 100 (#T9284, Sigma-Aldrich), then washed with PBS. Non-specific protein binding was blocked with 1% (v/v) Bovine serum albumin (BSA, #A2153, Sigma-Aldrich, Germany) for 20 min at RT. BMDMs were incubated with primary mouse polyclonal anti-histone H2A.X (#PA5-28778, Thermo Fisher Scientific, USA) antibody for 1 h at RT in 1:500 dilutions. BMDMs were washed and incubated with Alexa Fluor 488 goat anti-rabbit IgG (#A-11008, Thermo Fisher Scientific, USA) diluted 1:500 in 1% BSA at RT for 45 min in the dark. BMDMs were mounted with ProLong GOLD Anti-fade containing 4′,6-diamidino-2-phenylindole (DAPI). The slides were kept in the dark for 30 min at RT before sealing with nail polish and stored overnight at 4 °C in dark before analysis. The fluorescence imaging was performed with an Axiophot Zeiss microscope using a digital camera with *AxioVision 4.8* software. The obtained images were analysed by *Fiji ImageJ* software.

### mRNA and miRNA qRT-PCR

Total RNA including miRNAs was extracted from BMDMs using miRNAeasy Kit (#217004, Qiagen, Germany). The mRNA (2 μg) and miRNAs (100 ng) were separately reverse transcribed using Superscript III First-Strand synthesis system (#18080-51, Invitrogen, Germany) and miRNA universal cDNA synthesis kit II (#203301, Exiqon, Denmark) for reverse transcript PCR (RT-PCR) and subsequent real-time quantitative PCR (qRT-PCR). Detection of gene expression was performed with KapaFast-SYBR Green (#KAPBKK4606, Peqlab, Germany) and measurements were performed on a BioRad iCycler iQ Real-Time PCR Detection System (Bio-Rad Laboratories). The relative expression levels of mRNAs and miRNA were normalized to that of GAPDH and 5S rRNA, respectively^68^. The murine primers used to detect IL-1β, IL-2, IL4, IL-6, IL-10, IL-12, TNFα, IFNγ, TGF-β, and NOS2 expression are summarized in Table 1. For amplification of different miRNAs, hsa-miR-9-5p LNA PCR primer set (#204513), hsa-miR-10a-5p LNA TM PCR primer set (#204778), has-miR-99b-5p LNA PCR primer set (#205983), hsa-miR-146a-5p LNA PCR primer set (#204688), mmu-miR-155-5p LNA PCR primer set (#205930), and reference 5S rRNA primer set (#203906) were used and the reaction was set up as recommended by Exiqon^67,69^.

**Table 1:** Murine primer sequences.

Primer	Sequences
IL-1β	F 5^−^ TACCTGTGGCCTTGGGCCTCAA - 3^−^ R 5^−^ GCTTGGGATCCACACTCTCCAGC - 3^−^
IL-2	F 5^−^ AGGAACCTGAAACTCCCCAG - 3^−^ R 5^−^ CTTTCAATTCTGTGGCCTGCTT - 3^−^
IL-4	F 5^−^ AGGAGAAGGGACGCCATGCAC - 3^−^ R 5^−^ GCGAAGCACCTTGGAAGCCCTAC - 3^−^
IL-6	F 5^−^ TGGAGTCACAGAAGGAGTGGCTA - 3^−^ R 5^−^ TCTGACCACAGTGAGGAATGTCC - 3^−^
IL-10	F 5^−^ GGCGCTGTCATCGATTTCTCCCC - 3^−^ R 5^−^ GGCCTTGTAGACACCTTGGTCTT - 3^−^
IL-12	F 5^−^ AATCAGGGCTGCGAAGGTA - 3^−^ R 5^−^ AGGCCCTGGTTTCTTATCAA - 3^−^
TGF-β	F 5^−^ GAGCCCGAAGCGGACTACTA - 3^−^ R 5^−^ TGGTTTTCTCATAGATGGCGTTG - 3^−^
TNF-α	F 5^−^ ATAGCTCCCAGAAAAGCAAGC - 3^−^ R 5^−^ CACCCCGAAGTTCAGTAGACA - 3^−^
IFN-γ	F 5^−^ GGCTGTTACTGCCACGGCACA - 3^−^ R 5^−^ CACCATCCTTTTGCCAGTTCCTC - 3^−^
NOS2	F 5^−^ AGTCAACTGCAAGAGAACGGA - 3^−^ R 5^−^ TGAGAACAGCACAAGGGGTT - 3^−^

### Immunoblotting

BMDMs were stimulated and treated as indicated then washed once with PBS (#D8537, Sigma-Aldrich, Germany) and equal amounts of H_2_O and 2X Lammelli’s Buffer for cell lysis were added. Proteins were denatured at 95℃ for 7 min and stored at -20℃. Equal amounts of proteins (15 μg) were separated on 10% sodium dodecyl sulfate–polyacrylamide (SDS) gels and transferred to PVDF membranes (#10600023, Amersham Biosciences, UK). Nonspecific binding sites were blocked for 1 h at room temperature with 5% non-fat dry milk in Tris-buffered saline with 1% Tween. Membranes were probed with the indicated primary antibodies (all from Cell Signaling Technology and diluted 1:1000): anti- TLR2 (#13744s), TLR4 (#14358s), ERK1/2 (#9102s), pERK1/2 (Thr202/Tyr204, #9101s), SAPK/JNK (#9252s), pSAPK/JNK (Thr183/Tyr185, #4671s), p38 (#8690S), pp38 (Thr180/Tyr182, #4511s), IκBα (#4812s), pIκBα (Ser32, #2859S), AKT (#9272s), pAKT (T308, #2965s), mTOR (#2983s), phospho-mTOR (Ser2448, 2971s), and GAPDH (1:2000; #2118s) followed by HRP-conjugated secondary antibodies (#7074P2). Membranes were washed thrice and the antibodies visualized with enhanced chemiluminescent HRP substrate (#R-03031-D25 and R-03025-D25, advansta, USA). For detection of signals, X-ray films or versa doc were used. To confirm loading control after detection of phosphorylated protein, membranes were stripped using ReBlot Plus strong antibody stripping solution (#2504, Merck, Germany), blocked and re-probed with different antibodies against whole protein. Protein bands were quantified using *Image lab* & *ImageJ* software. Results are shown as the ratio of total protein to GAPDH normalized to the untreated control group. To assign the right protein size ProSieve QuadColor Protein Marker was used as a marker (6 μl, #00193837, Lonza).

### Enzyme linked immunosorbent assay (ELISA)

Supernatants from co-culture assays were analyzed for IL-1β (#88701322), IL-4 (#88704422), IL-6 (#88706422), IL-10 (#88710522), IL-12 (#88712188), TGF-β (#88835022), TNF-α (#88732422) and IFN-γ (#88831422) production via sandwich ELISA kits recommended by eBioscience manufacturer's instructions. Cytokines were quantified by comparison to recombinant standard proteins.

### Statistics

Given data are provided as means ± SEM. All data were tested for significance using unpaired Student’s t-test or ANOVA (one way & two ways). Data were analyzed by Excel 2010 or GraphPad Prism Software, USA. A value of *P* value ≤ 0.05 was statistically significant. For further details please see Supplementary Tables, where the significance of time and group factor are displayed for all experiments.

## Supplementary Information

Supplementary Information
