# Supplementary material for: The gut microbiota metabolite urolithin A inhibits NF-κB activation in LPS stimulated BMDMs
Source: Sci Rep. 2021 Mar 29;11:7117. doi: 10.1038/s41598-021-86514-6 (PMC8007722; doi:10.1038/s41598-021-86514-6)
Supplement: Supplementary file 1 — Supplementary Information [file 41598_2021_86514_MOESM1_ESM.pdf]

The gut microbiota metabolite urolithin A inhibits NF-κB activation in LPS stimulated BMDMs

Khalid N. M. Abdelazeem[1,2], M. Zaher Kalo[3], Sandra Beer-Hammer* [3] & Florian Lang[1]

[1]Department of Internal Medicine III, Eberhard Karls University of Tübingen, Tübingen, Germany

[2]Radiation Biology Research Department, National Centre for Radiation Research and Technology, Atomic Energy Authority, Cairo, Egypt,

[3]Department of Pharmacology, Experimental Therapy & Toxicology, Institute of Experimental and Clinical Pharmacology and Pharmacogenomic, University of Tübingen, Wilhelmstrasse 56, 72074 Tübingen, Germany

***Corresponding author:**

Professor Sandra Beer-Hammer, Department of Pharmacology, Experimental Therapy & Toxicology, Institute of Experimental and Clinical Pharmacology and Pharmacogenomic, University of Tübingen, Wilhelmstrasse 56, 72074 Tübingen, Germany

Telefon +49 7071 29-74594,
Telefax +49 7071 29-4942
E-Mail; sandra.beer-hammer@uni-tuebingen.de

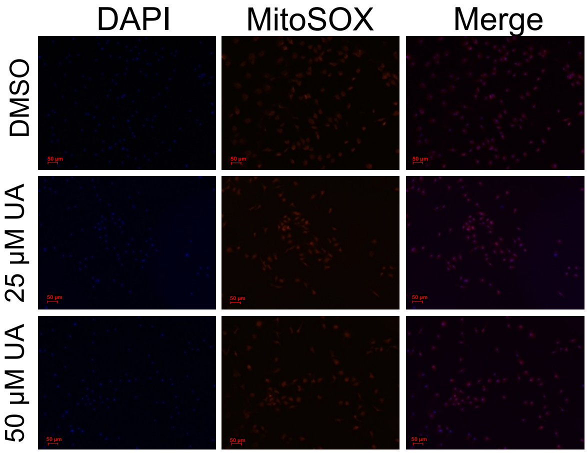

S Fig 1

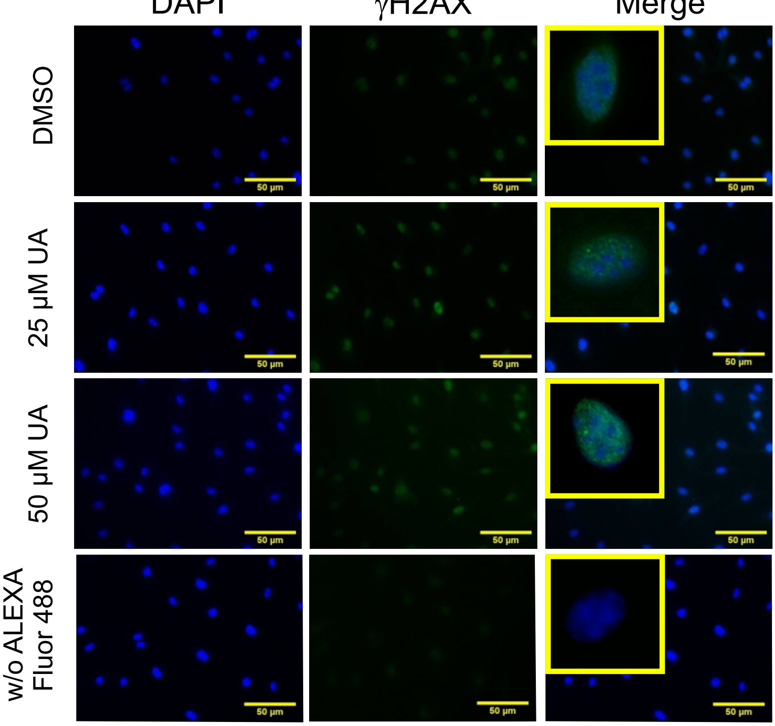

S Fig 2

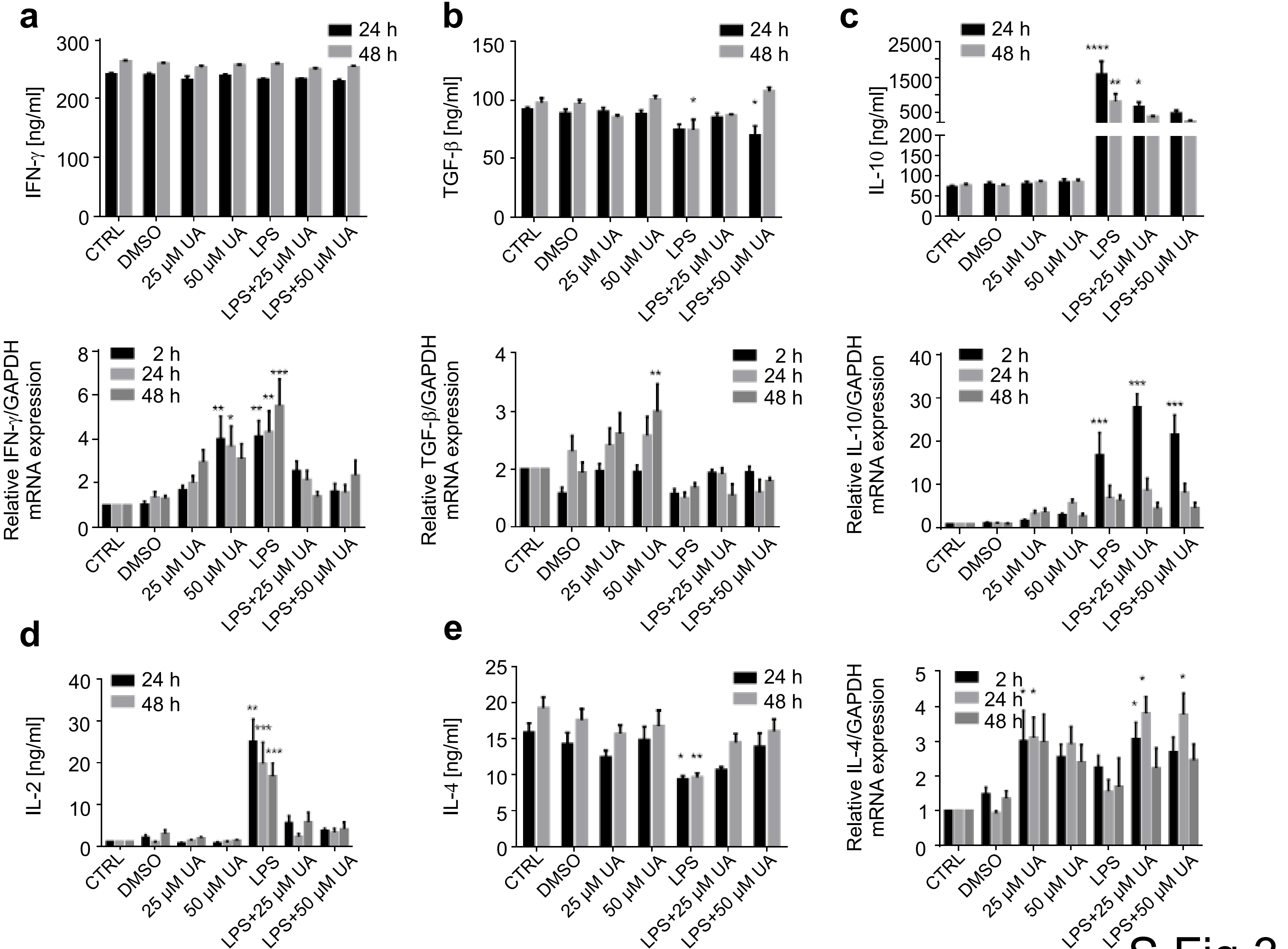
a
b
c
d
e
24 h
48 h
2 h
IFN-γ [ng/ml]
TGF-β [ng/ml]
IL-10 [ng/ml]
IL-2 [ng/ml]
IL-4 [ng/ml]
Relative IFN-γ/GAPDH mRNA expression
Relative TGF-β/GAPDH mRNA expression
Relative IL-10/GAPDH mRNA expression
Relative IL-4/GAPDH mRNA expression
CTRL
DMSO
25 μM UA
50 μM UA
LPS
LPS+25 μM UA
LPS+50 μM UA

S Fig 3

a

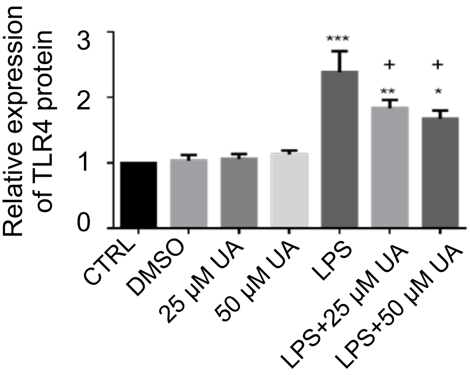

Relative expression of TLR4 protein
3
2
1
0
***
+
+
**
*
CTRL
DMSO
25 μM UA
50 μM UA
LPS
LPS+25 μM UA
LPS+50 μM UA

b

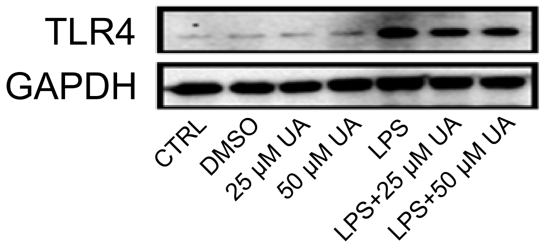

TLR4
GAPDH
CTRL
DMSO
25 μM UA
50 μM UA
LPS
LPS+25 μM UA
LPS+50 μM UA

S Fig 4

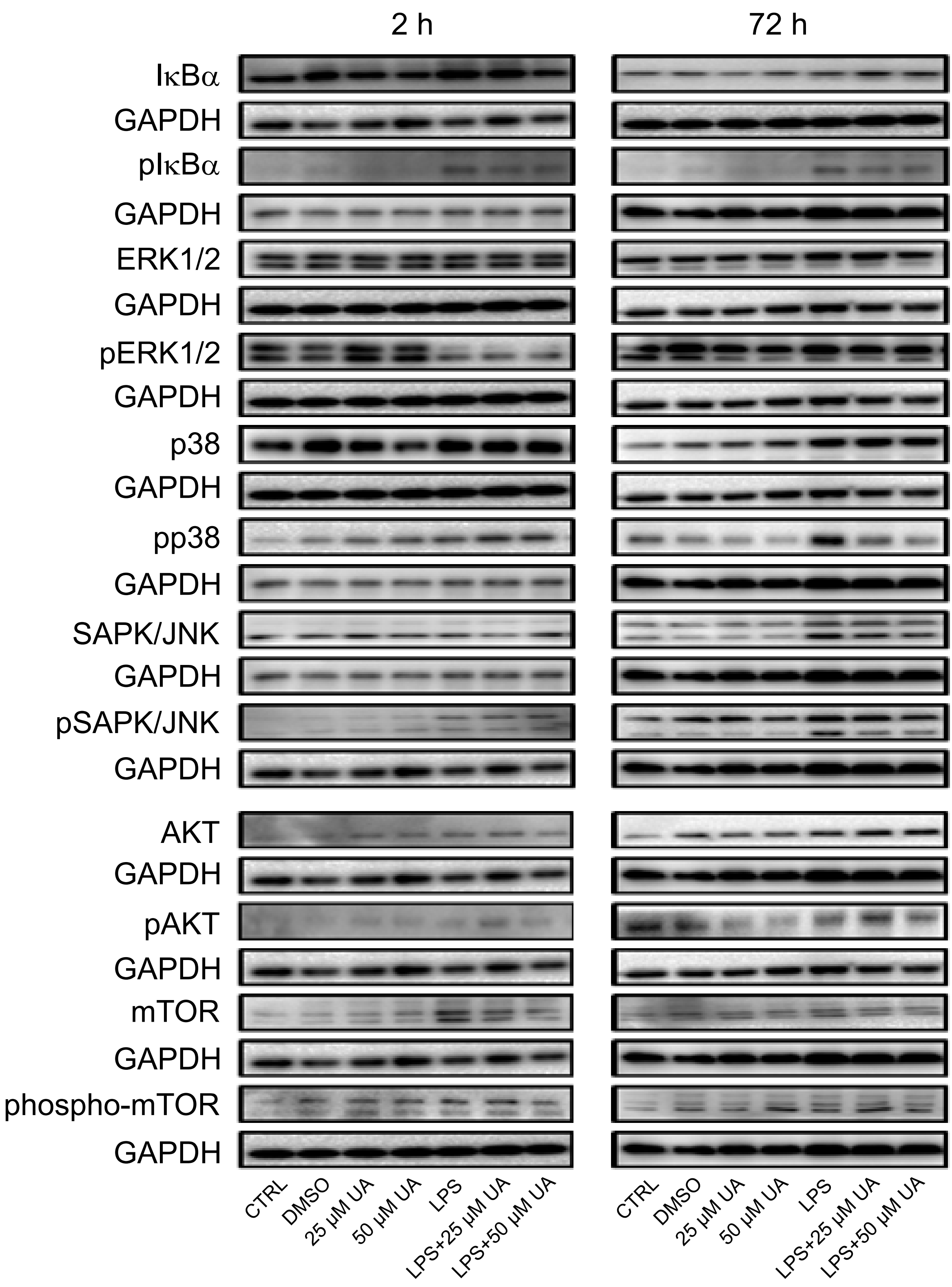
2 h
72 h
IκBα
GAPDH
pIκBα
GAPDH
ERK1/2
GAPDH
pERK1/2
GAPDH
p38
GAPDH
pp38
GAPDH
SAPK/JNK
GAPDH
pSAPK/JNK
GAPDH
AKT
GAPDH
pAKT
GAPDH
mTOR
GAPDH
phospho-mTOR
GAPDH
CTRL
DMSO
25 μM UA
50 μM UA
LPS
LPS+25 μM UA
LPS+50 μM UA

S Fig 5

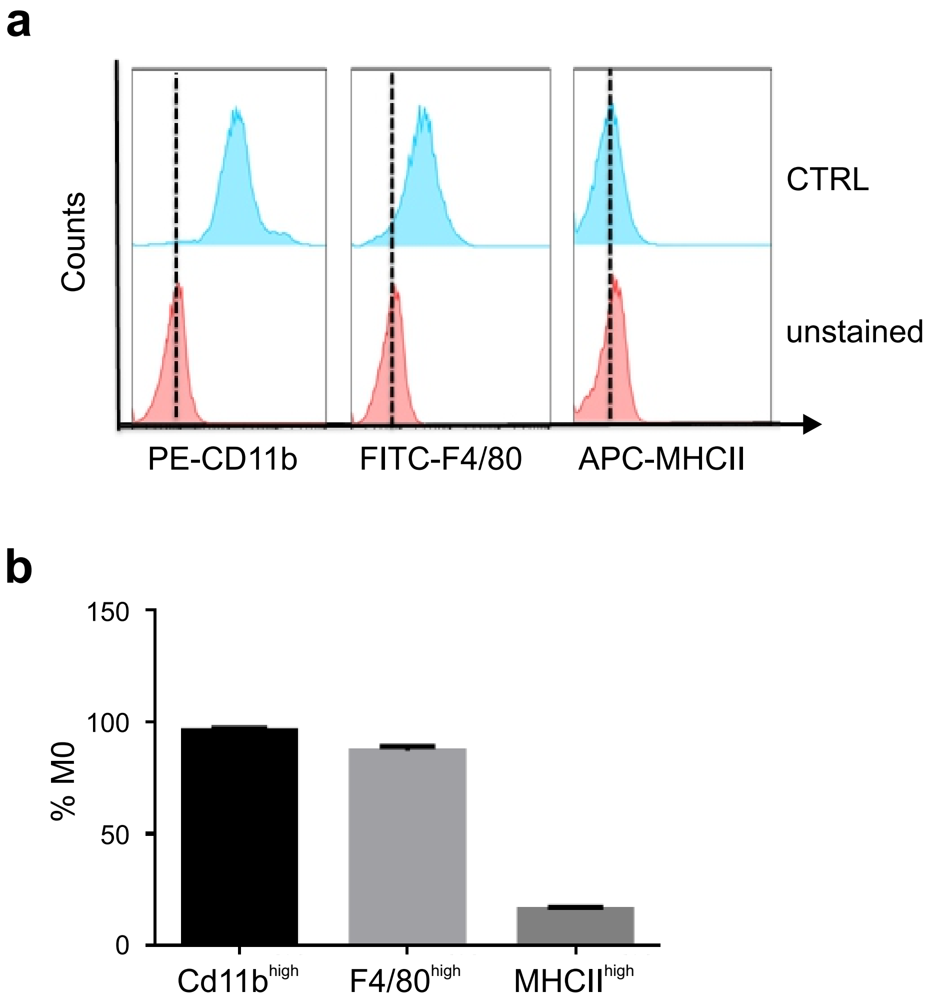
a
Counts
CTRL
unstained
PE-CD11b
FITC-F4/80
APC-MHCII
b
150
100
50
0
% M0
Cd11bhigh
F4/80high
MHCIIhigh

S Fig 6

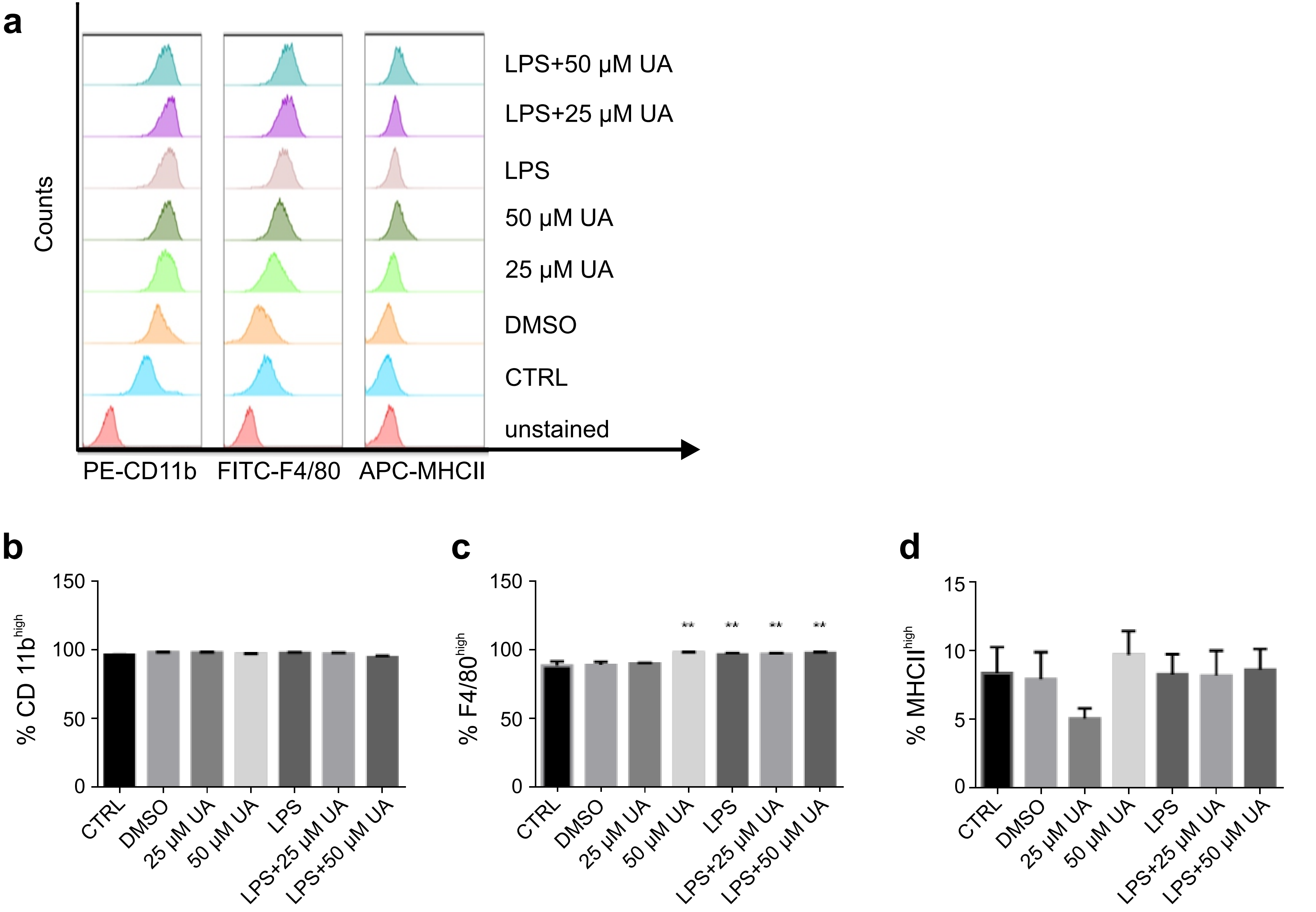
a
Counts
LPS+50 μM UA
LPS+25 μM UA
LPS
50 μM UA
25 μM UA
DMSO
CTRL
unstained
PE-CD11b
FITC-F4/80
APC-MHCII
b
% CD 11b^high
150
100
50
0
CTRL
DMSO
25 μM UA
50 μM UA
LPS
LPS+25 μM UA
LPS+50 μM UA
c
% F4/80^high
**
d
% MHCII^high
15
10
5
0

S Fig 7

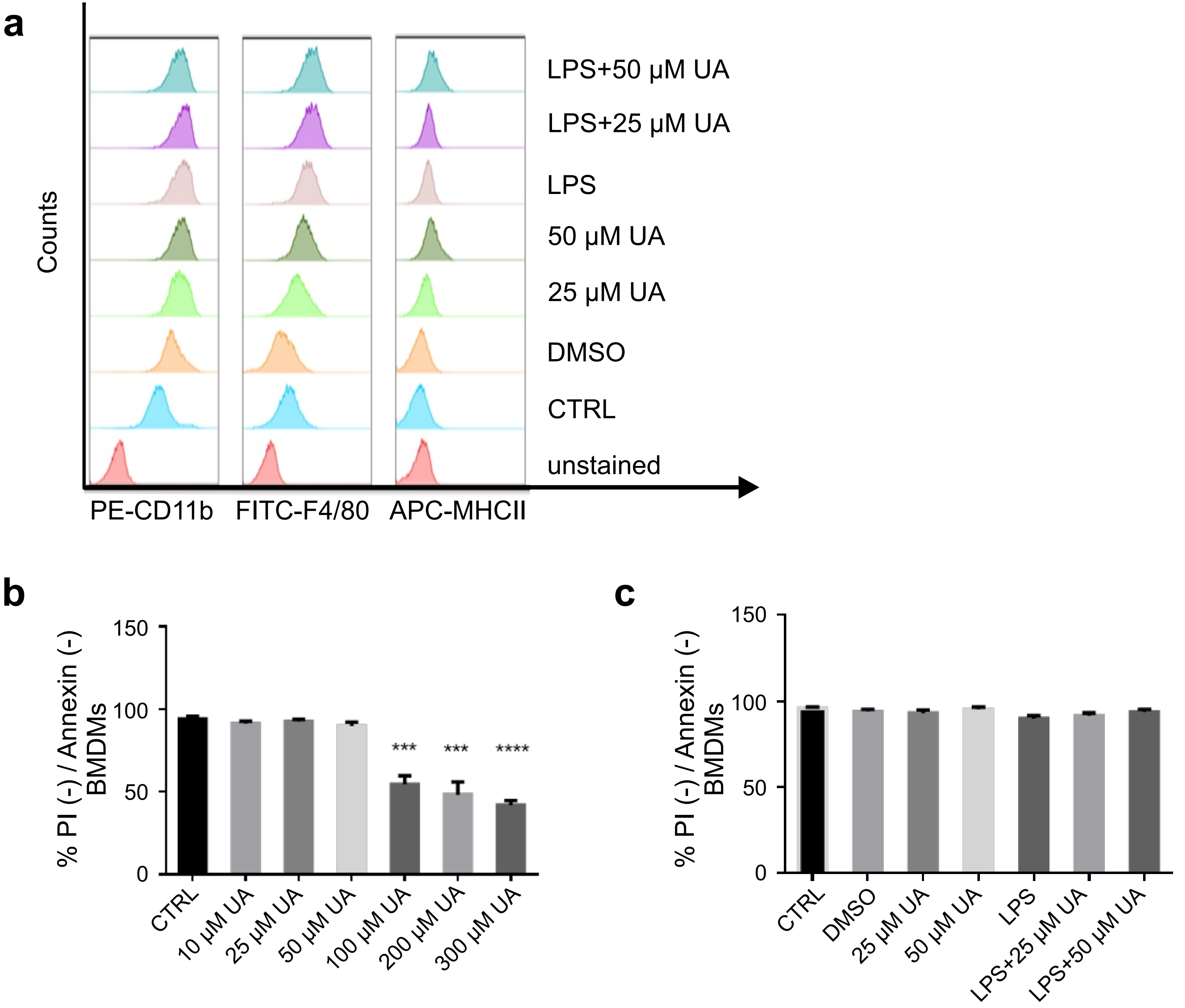
a
LPS+50 μM UA
LPS+25 μM UA
LPS
50 μM UA
25 μM UA
DMSO
CTRL
unstained
Counts
PE-CD11b
FITC-F4/80
APC-MHCII
b
% PI (-) / Annexin (-) BMDMs
150
100
50
0
***
***
****
CTRL
10 μM UA
25 μM UA
50 μM UA
100 μM UA
200 μM UA
300 μM UA
c
% PI (-) / Annexin (-) BMDMs
150
100
50
0
CTRL
DMSO
25 μM UA
50 μM UA
LPS
LPS+25 μM UA
LPS+50 μM UA

S Fig 8

Supplementary Figure Legends

Supplementary Figure 1 Effect of urolithin A or DMSO on superoxide production in murine BMDMs

Murine BMDMs were treated either by UA (25 µM or 50 µM) or DMSO for 48h. Both DMSO and UA did not record any observable changes. Nuclei were counterstained with DAPI (blue). Scale bar represents 50 µm. Murine BMDMs treated with DMSO were used as negative control. Abbreviations: DMSO, dimethyl sulfoxide; UA, urolithin A.

Supplementary Figure 2 Influence of urolithin A or DMSO on DSBs in murine BMDMs after 48h

Murine BMDMs were treated either by UA (25 µM or 50 µM) or DMSO for 48h. Both UA and DMSO did not register any significant effect on DSBs after 48h. Nuclei were counterstained with DAPI (blue). Scale bar represents 50 µm. BMDMs treated with DMSO were used as negative control. Abbreviations: DMSO, dimethyl sulfoxide; UA, urolithin A.

Supplementary Figure 3 Urolithin A reduces the inflammatory cytokine production and mRNA expression in LPS-stimulated murine BMDMs

Murine BMDMs were stimulated by 1µg/ml of LPS in the presence or absence of UA (25 µM or 50 µM). Subsequently, expression of the inflammatory cytokines IFN-γ (a), TGFβ (b), IL-10 (c), IL-2 (d), and IL-4 (e) were measured in BMDMs by qRT-PCR (over GAPDH) and ELISA at depicted time points. The unstimulated and untreated BMDMs were used as control. BMDMs treated with DMSO were used as negative control. Arithmetic means ± SEM from seven independent experiments are depicted. Two way ANOVA was used and * ($p < 0.05$), ** (P 0.01), *** ($p < 0.001$), and **** ($p < 0.0001$) indicate statistically significant differences compared to respective control. Abbreviations: DMSO, dimethyl sulfoxide; UA, urolithin A; LPS, lipopolysaccharides; IL, Interleukin; GAPDH, glyceraldehyde 3-phosphate dehydrogenase; IFN, interferon gamma; TGF, transforming growth factor.

Supplementary Figure 4 Sensitivity of TLR4 expressions to urolithin A (UA) in LPS-stimulated murine BMDMs

Murine BMDMs were stimulated by 1µg/ml of LPS in the presence or absence of UA (25 µM or 50 µM), then harvested and subjected to western blot after 72h. (a) Bar graph represents relative band intensities of TLR4 normalized to GAPDH. The unstimulated and untreated BMDMs were used as control. BMDMs treated with DMSO were used as negative control. (b) Representative image of TLR4 protein expression assessed by western blot analysis. Data are shown as means ± SEM from four independent experiments. One way ANOVA was used and * ($p < 0.05$), ** ($p < 0.001$), and *** ($p < 0.001$) indicate statistically significant differences compared to untreated control. + ($p < 0.05$) indicate statistically significant differences compared to LPS. Abbreviations: DMSO, dimethyl sulfoxide; LPS, lipopolysaccharides; UA, urolithin A; TLR, Toll like receptor; GAPDH, glyceraldehyde-3- phosphate dehydrogenase.

Supplementary Figure 5 Effect of urolithin A on IκBα, ERK1/2, p38, SAPK/JNK, AKT and mTOR expression and phosphorylation in LPS-stimulated murine BMDMs

Murine BMDMs were stimulated by 1µg/ml of LPS in the presence or absence of UA (25 µM or 50 µM) and harvested at 2h and 72h followed by western blot analysis. (a) Phosphorylation of IκBα, ERK1/2, and AKT were monitored by immunoblot using pIκBα (Ser32/36) monoclonal antibodies, phospho-p44/42 MAP kinase (Thr202/Tyr204) polyclonal antibodies, and pAKT (Ser473) monoclonal antibodies. Subsequently, blots were stripped and re-incubated with antibodies against total IκBα, ERK1/2 and AKT. (b) Phosphorylation of p38, SAPK/JNK, and mTOR were monitored by immunoblot using phospho-p38 MAP kinase (Thr180/Tyr182), and phospho-SAPK/JNK MAP kinase (Thr183/Tyr185) and phospho-mTOR (Ser2448) monoclonal antibodies, respectively at depicted time points. Subsequently, blots of the same membrane were stripped and re-incubated with antibody against total p38, SAPK/JNK and mTOR. GAPDH served as a loading control. The unstimulated and untreated BMDMs were used as control. BMDMs treated with DMSO were used as negative control. Abbreviations: DMSO, dimethyl sulfoxide; LPS, lipopolysaccharides; UA, urolithin A; GAPDH, glyceraldehyde-3- phosphate dehydrogenase.

Supplementary Figure 6 FACS phenotyping of BMDMs

Naive bone marrow cells were isolated and subsequently cultured for one week in DMEM complete media (M0) and then analyzed for macrophage markers. (a) FACS histogram displaying the harvested BMDMs (M0) which were defined as F4/80^{high}, CD11b^{high} and MHCII^{high}. (b) Arithmetic means ± SEM (n = 5) of percentage of the positive gated populations of M0.

Supplementary Figure 7 FACS phenotyping of BMDMs after 72h of stimulation

Naive bone marrow cells were isolated and subsequently cultured for one week in DMEM complete media and then stimulated with 1µg/ml LPS in the presence or absence of UA (25 µM or 50 µM). (a) FACS histogram displaying the expression of macrophage marker after 72h of desired treatment using CD11b, F4/80 and MHCII. Arithmetic means ± SEM (n = 5) of percentage of the positive gated populations of stimulated macrophages which were defined as CD11b^{high} (b), F4/80^{high} (c) and MHCII^{high} (d). * ($p<0.05$) and ** ($P<0.001$) indicates statistically significant difference compared to control. Abbreviations: DMSO, dimethyl sulfoxide; UA, urolithin A; RAD, radiation.

Supplementary Figure 8 Influence of urolithin A on the viability of LPS-stimulated murine BMDMs after 72h

Naive bone marrow cells were isolated and subsequently cultured for one week in DMEM complete media and then stimulated with 1µg/ml LPS in the presence or absence of UA (25 µM or 50 µM). (a) Dot plots of mature BMDMs which were treated as indicated. After 72h the cell viability was measured by Annexin-V /PI compared to untreated control cells. (b) Arithmetic means ± SEM (n = 5) of BMDMs which were treated with different concentrations of UA for 72h. (c) Values show the percentage of live BMDMs Annexin-V$^-$/PI$^-$ after indicated treatments. Arithmetic means ± SEM (n = 5). *** ($P<0.001$) and **** ($P<0.0001$) indicate statistically significant differences compared to control. Abbreviations: DMSO, dimethyl sulfoxide; UA, urolithin A; LPS, lipopolysaccharides.

SUPPLEMENTARY TABLES

Supplementary Table 1 The source of variation in intracellular calcium in LPS-stimulated or X-irradiated murine BMDMs

Source of variation	% of total variation	F(DFn, DFd)	P value
Interaction	7.747	F (13, 145) = 1.645	P = 0.0792
Group factor	33.64	F (13, 145) = 7.144	P < 0.0001
Time factor	4.312	F (1, 145) = 11.91	P = 0.0007

Supplementary Table 2 The source of variation in γH2AX phosphorylation in LPS-stimulated or X-irradiated murine BMDMs

Source of variation	% of total variation	F (DFn, DFd)	P value
Interaction	6.728	F (13, 318) = 2.730	P = 0.0011
Group factor	32.27	F (13, 318) = 13.09	P < 0.0001
Time factor	0.4730	F (1, 318) = 2.495	P = 0.1152

Supplementary Table 3 The source of variation in protein expression of TLR2 in LPS-stimulated or X-irradiated murine BMDMs

Source of variation / TLR2	% of total variation	F (DFn, DFd)	P value	P value summary
Interaction	15.61	F (36, 507) = 5.298	P < 0.0001	****
Group factor	39.95	F (12, 507) = 40.67	P < 0.0001	****
Time factor	1.481	F (3, 507) = 6.032	P = 0.0005	***

Supplementary Table 4 The source of variation in protein expression and phosphorylation of ERK1/2 in LPS-stimulated or X-irradiated murine BMDMs

Source of variation / pERK1/2 over GAPDH	% of total variation	F (DFn, DFd)	P value	P value summary
Interaction	42.83	F (36, 286) = 7.898	P < 0.0001	****
Group factor	13.38	F (12, 286) = 7.402	P < 0.0001	****
Time factor	0.5391	F (3, 286) = 1.193	P = 0.3126	ns
**Source of variation / ERK1/2 over GAPDH**	**% of total variation**	**F (DFn, DFd)**	**P value**	**P value summary**
Interaction	20.60	F (36, 386) = 4.361	P < 0.0001	****
Group factor	29.42	F (12, 386) = 18.68	P < 0.0001	****
Time factor	2.287	F (3, 386) = 5.808	P = 0.0007	***
**Source of variation / pERK1/2 over ERK**	**% of total variation**	**F (DFn, DFd)**	**P value**	**P value summary**
Interaction	31.32	F (36, 260) = 3.921	P < 0.0001	****
Group factor	9.705	F (12, 260) = 3.645	P < 0.0001	****
Time factor	1.286	F (3, 260) = 1.931	P = 0.1249	ns

Supplementary Table 5 The source of variation in protein expression and phosphorylation of MAPK p38 in LPS-stimulated or X-irradiated murine BMDMs

Source of variation / pp38 over GAPDH	% of total variation	F (DFn, DFd)	P value	P value summary
Interaction	19.15	F (36, 351) = 4.448	P < 0.0001	****
Group factor	26.46	F (12, 351) = 18.44	P < 0.0001	****
Time factor	12.37	F (3, 351) = 34.48	P < 0.0001	****
**Source of variation / p38 over GAPDH**	**% of total variation**	**F (DFn, DFd)**	**P value**	**P value summary**
Interaction	27.15	F (36, 247) = 5.127	P < 0.0001	****
Group factor	37.25	F (12, 247) = 21.10	P < 0.0001	****
Time factor	1.456	F (3, 247) = 3.299	P = 0.0211	*
**Source of variation / phospho/p38 over p38**	**% of total variation**	**F (DFn, DFd)**	**P value**	**P value summary**
Interaction	29.43	F (36, 247) = 5.008	P < 0.0001	****
Group factor	18.45	F (12, 247) = 9.419	P < 0.0001	****
Time factor	10.60	F (3, 247) = 21.65	P < 0.0001	****

Supplementary Table 6 The source of variation in protein expression and phosphorylation of MAPK SAPK/JNK in LPS-stimulated or X-irradiated murine BMDMs

Source of variation / phospho-SAPK/JNK over GAPDH	% of total variation	F (DFn, DFd)	P value	P value summary
Interaction	15.06	F (36, 273) = 3.122	P < 0.0001	****
Group factor	45.48	F (12, 273) = 28.28	P < 0.0001	****
Time factor	0.4262	F (3, 273) = 1.060	P = 0.3665	ns
**Source of variation / SAPK/JNK over GAPDH**	**% of total variation**	**F (DFn, DFd)**	**P value**	**P value summary**
Interaction	6.266	F (36, 286) = 1.394	P = 0.0740	ns
Group factor	53.30	F (12, 286) = 35.57	P < 0.0001	****
Time factor	2.454	F (3, 286) = 6.551	P = 0.0003	***
**Source of variation / pSAPK/JNK over SAPK/JNK**	**% of total variation**	**F (DFn, DFd)**	**P value**	**P value summary**
Interaction	20.37	F (36, 208) = 1.877	P = 0.0034	**
Group factor	13.73	F (12, 208) = 3.795	P < 0.0001	****
Time factor	3.194	F (3, 208) = 3.531	P = 0.0158	*

Supplementary Table 7 The source of variation in protein expression of IκBα expression and phosphorylation in LPS-stimulated or X-irradiated murine BMDMs

Source of variation / pIκBα over GAPDH	% of total variation	F (DFn, DFd)	P value	P value summary
Interaction	3.277	F (36, 208) = 0.7886	P = 0.8001	ns
Group factor	71.94	F (12, 208) = 51.93	P < 0.0001	****
Time factor	0.7713	F (3, 208) = 2.227	P = 0.0861	ns
**Source of variation / IκBα over GAPDH**	**% of total variation**	**F (DFn, DFd)**	**P value**	**P value summary**
Interaction	19.38	F (36, 208) = 4.482	P < 0.0001	****
Group factor	46.84	F (12, 208) = 32.49	P < 0.0001	****
Time factor	8.799	F (3, 208) = 24.42	P < 0.0001	****
**Source of variation / pIκBα over IκBα**	**% of total variation**	**F (DFn, DFd)**	**P value**	**P value summary**
Interaction	16.26	F (36, 208) = 2.714	P < 0.0001	****
Group factor	45.24	F (12, 208) = 22.65	P < 0.0001	****
Time factor	3.884	F (3, 208) = 7.780	P < 0.0001	****

Supplementary Table 8 The source of variation in protein expression and phosphorylation of AKT in LPS-stimulated or X-irradiated murine BMDMs

Source of variation / pAKT over GAPDH	% of total variation	F (DFn, DFd)	P value	P value summary
Interaction	29.06	F (36, 299) = 5.809	P < 0.0001	****
Group factor	22.32	F (12, 299) = 13.39	P < 0.0001	****
Time factor	8.092	F (3, 299) = 19.41	P < 0.0001	****
**Source of variation / AKT over GAPDH**	**% of total variation**	**F (DFn, DFd)**	**P value**	**P value summary**
Interaction	15.74	F (36, 312) = 4.524	P < 0.0001	****
Group factor	46.02	F (12, 312) = 39.68	P < 0.0001	****
Time factor	8.075	F (3, 312) = 27.84	P < 0.0001	****
**Source of variation / pAKT over AKT**	**% of total variation**	**F (DFn, DFd)**	**P value**	**P value summary**
Interaction	25.51	F (36, 299) = 5.331	P < 0.0001	****
Group factor	15.43	F (12, 299) = 9.674	P < 0.0001	****
Time factor	19.95	F (3, 299) = 50.05	P < 0.0001	****

Supplementary Table 9 The source of variation in protein expression and phosphorylation of mTOR in LPS-stimulated or X-irradiated murine BMDMs

Source of variation / phospho-mTOR over GAPDH	% of total variation	F (DFn, DFd)	P value	P value summary
Interaction	15.42	F (36, 387) = 3.499	P < 0.0001	****
Group factor	30.73	F (12, 387) = 20.92	P < 0.0001	****
Time factor	6.764	F (3, 387) = 18.41	P < 0.0001	****
**Source of variation / mTOR over GAPDH**	**% of total variation**	**F (DFn, DFd)**	**P value**	**P value summary**
Interaction	13.69	F (36, 351) = 3.984	P < 0.0001	****
Group factor	50.05	F (12, 351) = 43.70	P < 0.0001	****
Time factor	3.277	F (3, 351) = 11.44	P < 0.0001	****
**Source of variation / phospho-mTOR over mTOR**	**% of total variation**	**F (DFn, DFd)**	**P value**	**P value summary**
Interaction	27.85	F (36, 351) = 7.738	P < 0.0001	****
Group factor	24.93	F (12, 351) = 20.78	P < 0.0001	****
Time factor	14.17	F (3, 351) = 47.24	P < 0.0001	****

Supplementary Table 10 The source of variation of pro-inflammatory cytokine mRNA expression in LPS-stimulated or X-irradiated murine BMDMs

Source of variation / IL-1β	% of total variation	F (DFn, DFd)	P value	P value summary
Interaction	24.47	F (26, 166) = 49.58	P < 0.0001	****
Group factor	68.95	F (13, 166) = 279.4	P < 0.0001	****
Time factor	3.339	F (2, 166) = 87.96	P < 0.0001	****
**Source of variation / IL-2**	**% of total variation**	**F (DFn, DFd)**	**P value**	**P value summary**
Interaction	3.335	F (26, 192) = 0.8858	P = 0.6283	ns
Group factor	66.63	F (13, 192) = 35.40	P < 0.0001	****
Time factor	1.049	F (2, 192) = 3.624	P = 0.0285	*
**Source of variation / IL-6**	**% of total variation**	**F (DFn, DFd)**	**P value**	**P value summary**
Interaction	44.60	F (26, 252) = 51.81	P < 0.0001	****
Group factor	38.68	F (13, 252) = 89.86	P < 0.0001	****
Time factor	8.371	F (2, 252) = 126.4	P < 0.0001	****
**Source of variation / IL-12**	**% of total variation**	**F (DFn, DFd)**	**P value**	**P value summary**
Interaction	21.03	F (26, 230) = 4.485	P < 0.0001	****
Group factor	37.76	F (13, 230) = 16.11	P < 0.0001	****
Time factor	4.157	F (2, 230) = 11.53	P < 0.0001	****
**Source of variation / TNF-α**	**% of total variation**	**F (DFn, DFd)**	**P value**	**P value summary**
Interaction	36.46	F (26, 246) = 34.51	P < 0.0001	****
Group factor	46.95	F (13, 246) = 88.90	P < 0.0001	****
Time factor	6.413	F (2, 246) = 78.93	P < 0.0001	****

Supplementary Table 11 The source of variation in anti-inflammatory cytokine mRNA expression in LPS-stimulated or X-irradiated murine BMDMs

Source of variation / IL-4	% of total variation	F (DFn, DFd)	P value	P value summary
Interaction	23.18	F (26, 259) = 6.454	P < 0.0001	****
Group factor	30.00	F (13, 259) = 16.71	P < 0.0001	****
Time factor	11.90	F (2, 259) = 43.07	P < 0.0001	****
**Source of variation / IL-10**	**% of total variation**	**F (DFn, DFd)**	**P value**	**P value summary**
Interaction	23.35	F (26, 235) = 4.678	P < 0.0001	****
Group factor	24.39	F (13, 235) = 9.770	P < 0.0001	****
Time factor	6.672	F (2, 235) = 17.37	P < 0.0001	****
**Source of variation / TGF-β**	**% of total variation**	**F (DFn, DFd)**	**P value**	**P value summary**
Interaction	2.195	F (26, 248) = 0.4064	P = 0.9960	ns
Group factor	46.23	F (13, 248) = 17.12	P < 0.0001	****
Time factor	0.05814	F (2, 248) = 0.1399	P = 0.8695	ns
**Source of variation / IFN-γ**	**% of total variation**	**F (DFn, DFd)**	**P value**	**P value summary**
Interaction	3.315	F (26, 239) = 0.5017	P = 0.9808	ns
Group factor	33.54	F (13, 239) = 10.15	P < 0.0001	****
Time factor	0.05949	F (2, 239) = 0.1170	P = 0.8896	ns
**Source of variation / NOS2**	**% of total variation**	**F (DFn, DFd)**	**P value**	**P value summary**
Interaction	27.23	F (26, 243) = 103.5	P < 0.0001	****
Group factor	68.19	F (13, 243) = 518.4	P < 0.0001	****
Time factor	1.943	F (2, 243) = 95.99	P < 0.0001	****

Supplementary Table 12 The source of variation of pro-inflammatory cytokines production in LPS-stimulated or X-irradiated murine BMDMs

Source of variation / IL-1β	% of total variation	F (DFn, DFd)	P value	P value summary
Interaction	10.39	F (12, 156) = 5.966	P < 0.0001	****
Group factor	66.12	F (12, 156) = 37.96	P < 0.0001	****
Time factor	0.8422	F (1, 156) = 5.802	P = 0.0172	*
**Source of variation / IL-6**	**% of total variation**	**F (DFn, DFd)**	**P value**	**P value summary**
Interaction	2.344	F (13, 168) = 1.764	P = 0.0525	ns
Group factor	80.47	F (13, 168) = 60.56	P < 0.0001	****
Time factor	0.01169	F (1, 168) = 0.1144	P = 0.7356	ns
**Source of variation / IL-12**	**% of total variation**	**F (DFn, DFd)**	**P value**	**P value summary**
Interaction	2.439	F (13, 166) = 0.5166	P = 0.9122	ns
Group factor	35.28	F (13, 166) = 7.472	P < 0.0001	****
Time factor	1.956	F (1, 166) = 5.386	P = 0.0215	*
**Source of variation / TNF-α**	**% of total variation**	**F (DFn, DFd)**	**P value**	**P value summary**
Interaction	16.29	F (13, 168) = 29.95	P < 0.0001	****
Group factor	73.38	F (13, 168) = 134.9	P < 0.0001	****
Time factor	3.299	F (1, 168) = 78.83	P < 0.0001	****

Supplementary Table 13 The source of variation of anti-inflammatory cytokines production in LPS-stimulated or X-irradiated BMDMs

**Source of variation/ IL-4**	**% of total variation**	**F (DFn, DFd)**	**P value**	**P value summary**
Interaction	7.409	F (13, 168) = 1.965	P = 0.0265	*
Group factor	33.41	F (13, 168) = 8.861	P < 0.0001	****
Time factor	10.46	F (1, 168) = 36.07	P < 0.0001	****
**Source of variation/ IL-10**	**% of total variation**	**F (DFn, DFd)**	**P value**	**P value summary**
Interaction	7.302	F (13, 168) = 3.094	P = 0.0004	***
Group factor	60.59	F (13, 168) = 25.68	P < 0.0001	****
Time factor	1.609	F (1, 168) = 8.866	P = 0.0033	**
**Source of variation/ TGF-β**	**% of total variation**	**F (DFn, DFd)**	**P value**	**P value summary**
Interaction	17.89	F (13, 168) = 3.691	P < 0.0001	****
Group factor	15.77	F (13, 168) = 3.253	P = 0.0002	***
Time factor	3.698	F (1, 168) = 9.917	P = 0.0019	**
**Source of variation/ IFN-γ**	**% of total variation**	**F (DFn, DFd)**	**P value**	**P value summary**
Interaction	4.273	F (13, 168) = 2.040	P = 0.0203	*
Group factor	27.07	F (13, 168) = 12.92	P < 0.0001	****
Time factor	41.59	F (1, 168) = 258.1	P < 0.0001	****

Supplementary western blots (whole bands)

In this project only **the first seven bands** were used and the other bands belong to another project.

- Prosieve ladder 300 kda (Lonza) was used (see image below).
- Usually the membrane was cut at 70 kda and the upper part was used for TLR4, mTOR, phospho-mTOR and the lower part for the other depicted proteins.
- The membrane was first incubated with antibodies against the phospho-protein, then stripped and controlled, blocked and incubated with the antibodies against the total protein.
- Either phospho- or total proteins were controlled to their corresponding GAPDH.

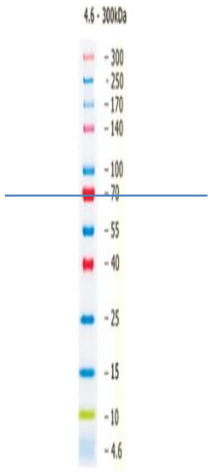

A

1- TLR4

72h	
TLR4	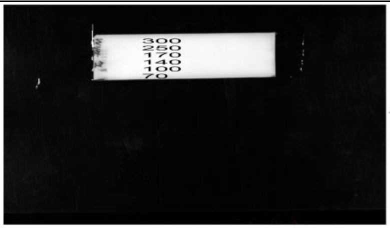 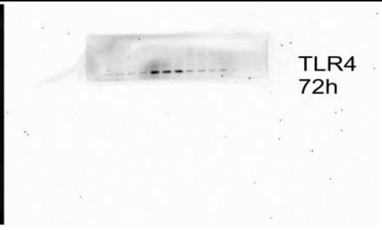
GAPDH	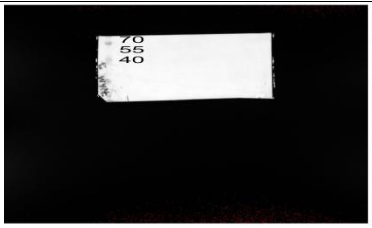 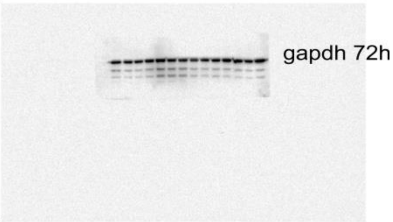

2- ERK1/2

2h	ERK1/2	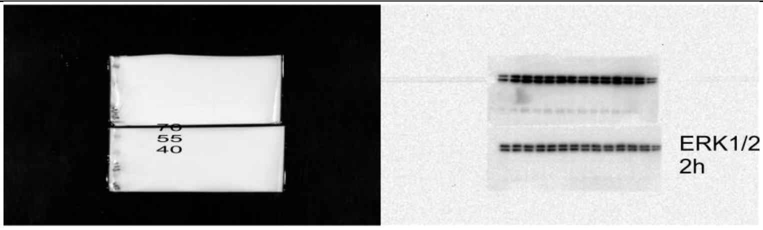
		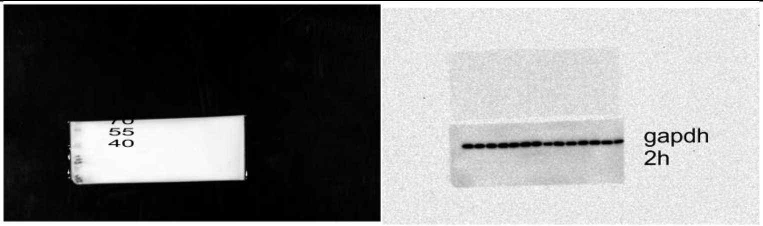
	pERK1/2	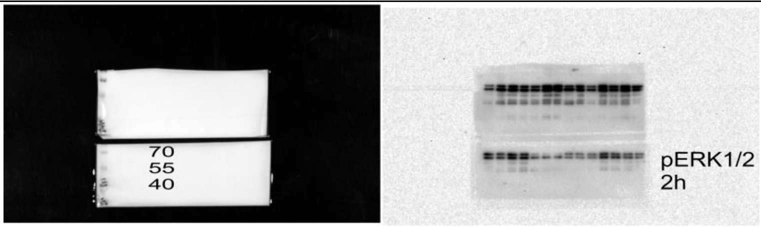
	GAPDH	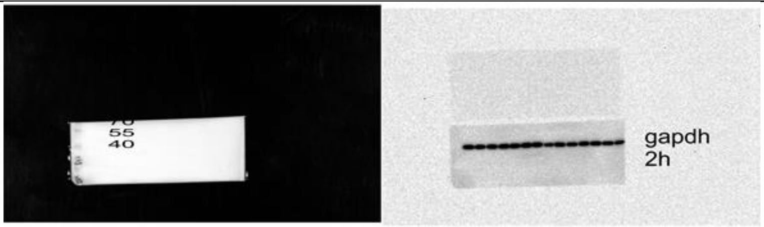
24h		
	ERK1/2	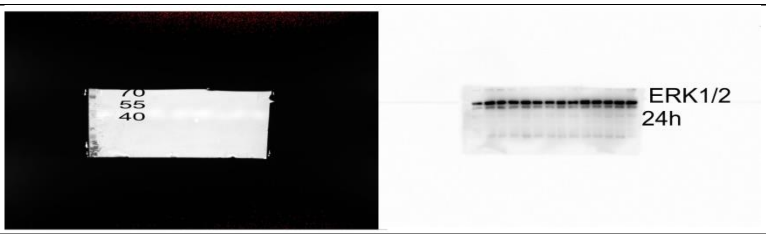
	pERK1/2	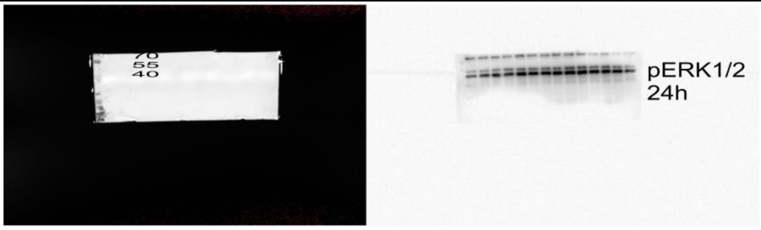
	GAPDH	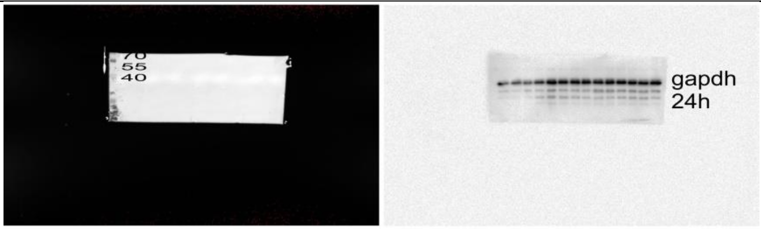

48h		
	ERK1/2	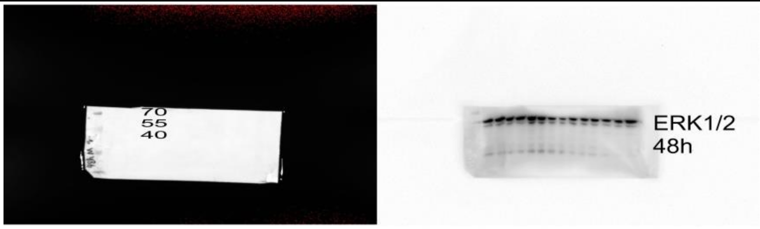
	pERK1/2	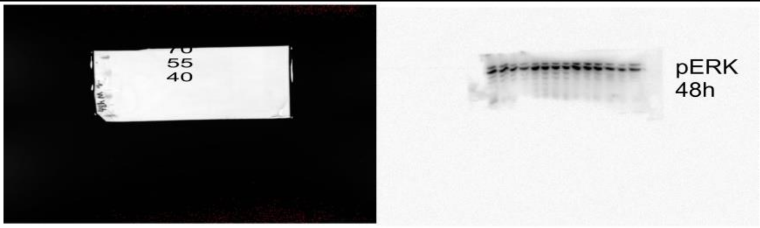
	GAPDH	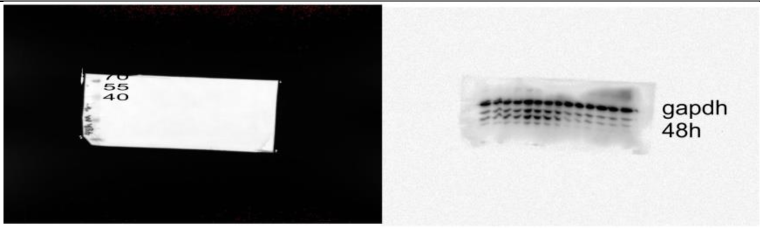
72h		
	ERK1/2	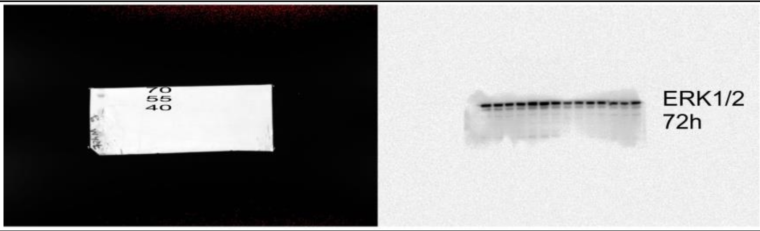
	pERK1/2	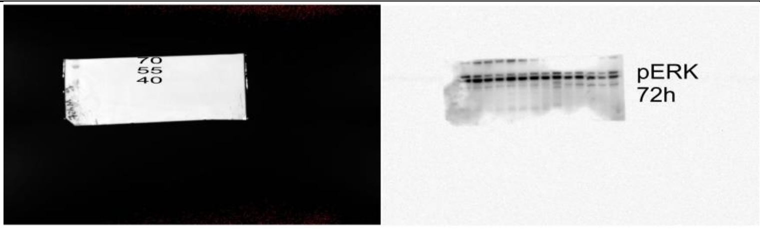
	GAPDH	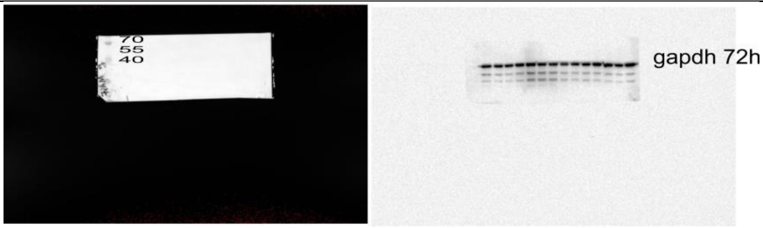

3- p38

2h		
	p38	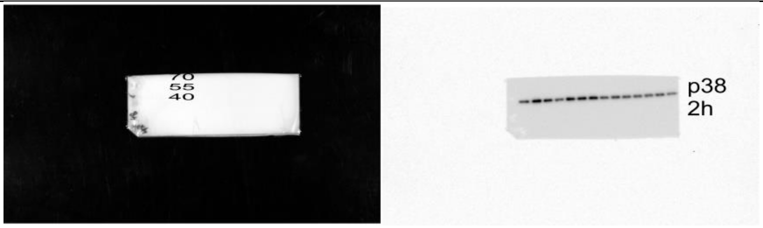
	GAPDH	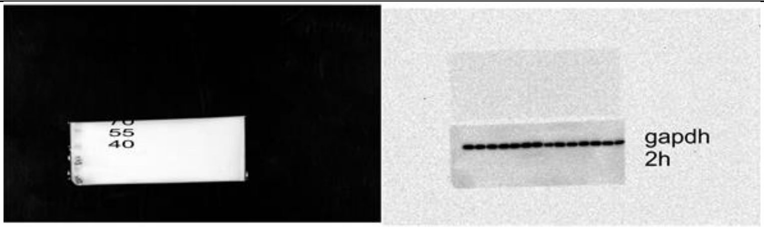
	pp38	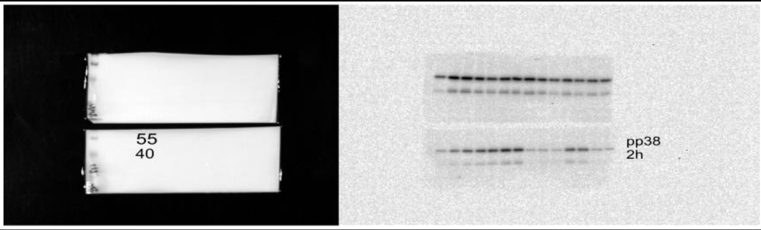
	GAPDH	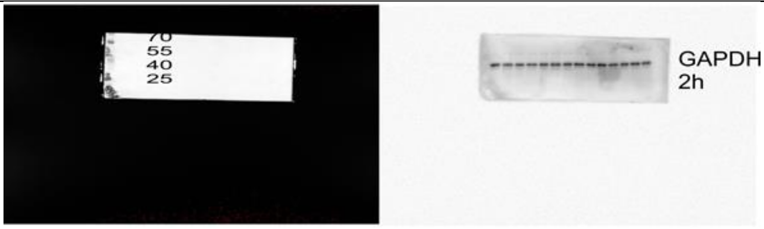
24h		
	p38	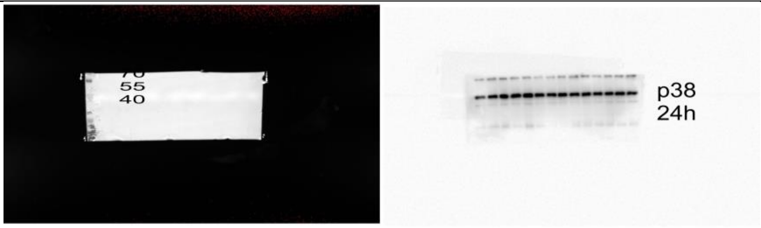
	GAPDH	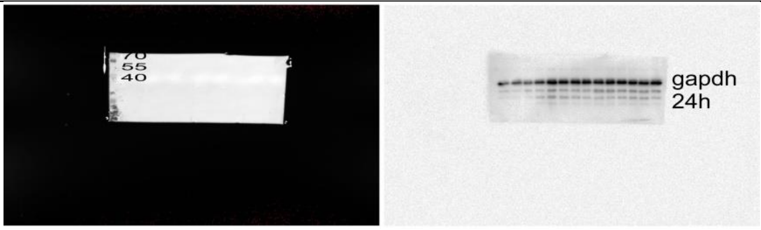

	pp38	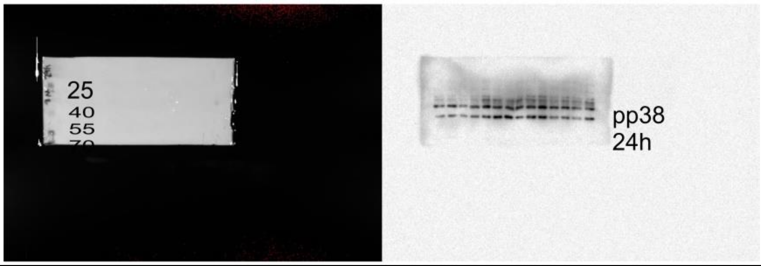
	GAPDH	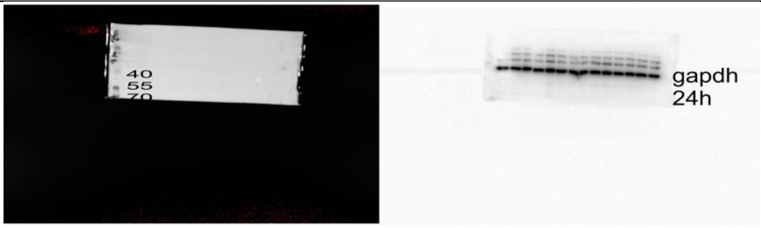
48h		
	p38	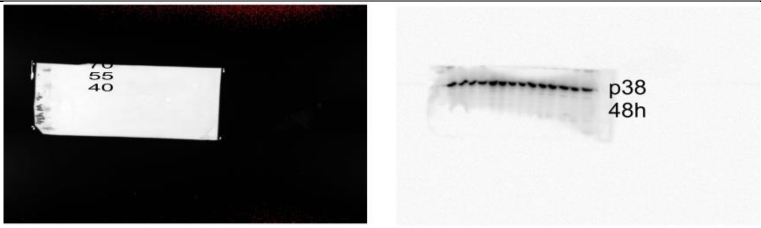
	GAPDH	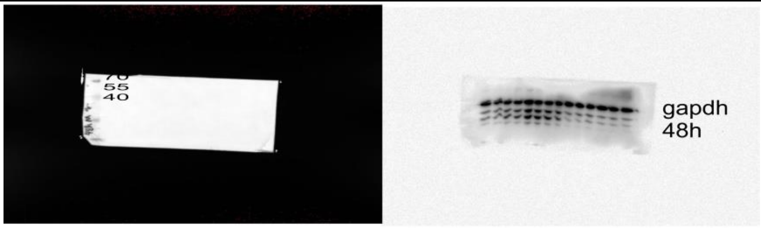
	pp38	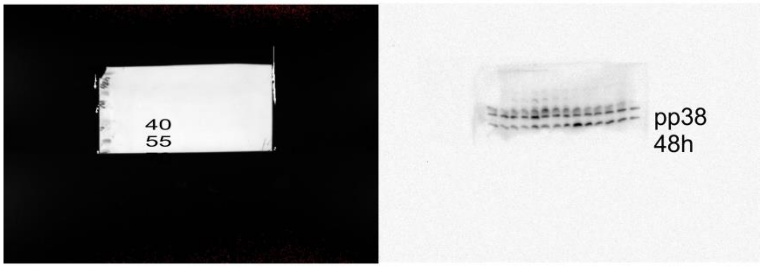
	GAPDH	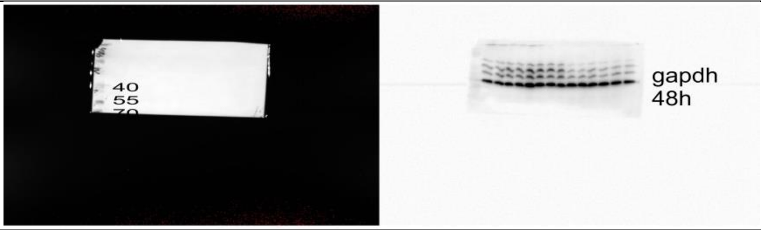
72h		

	p38	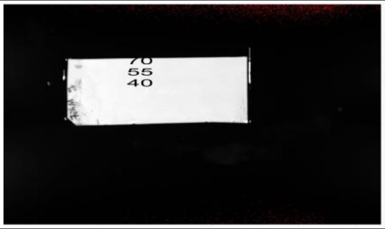 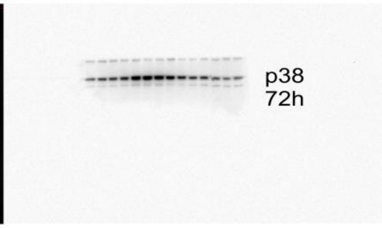
	GAPDH	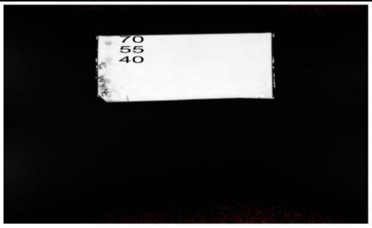 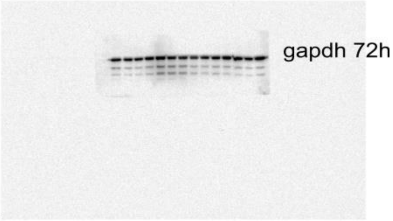
	pp38	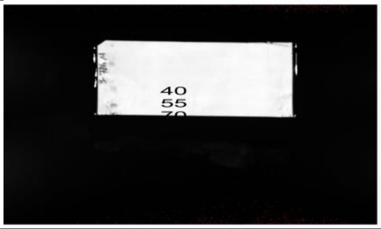 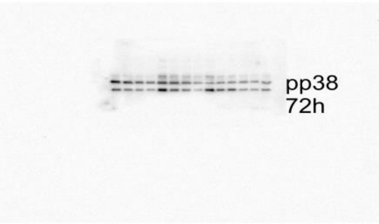
	GAPDH	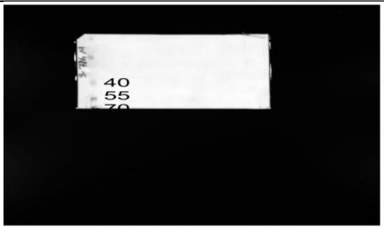 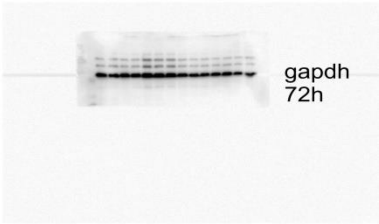

4- SAPK/JNK

2h	SAPK/JNK	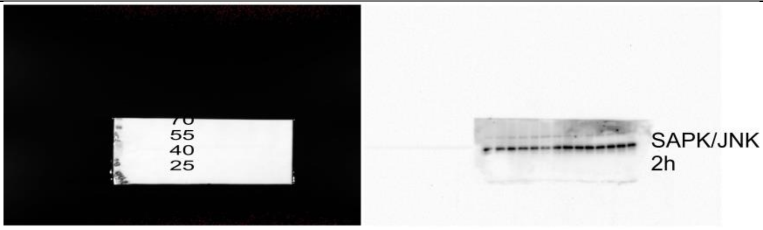
	GAPDH	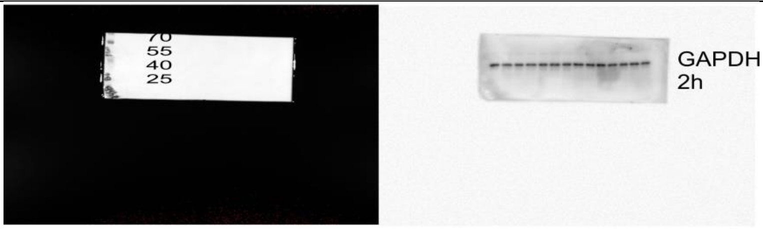
	pSAPK/JNK	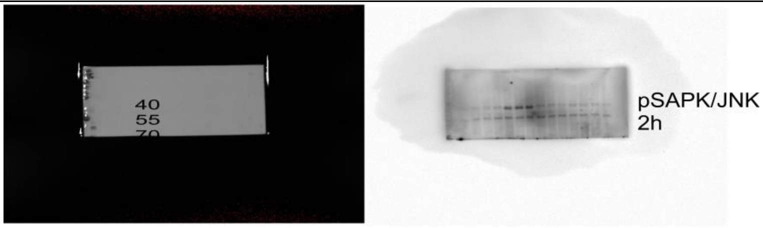
	GAPDH	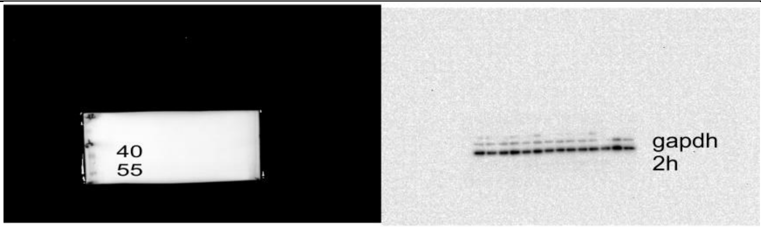
24h		
	SAPK/JNK	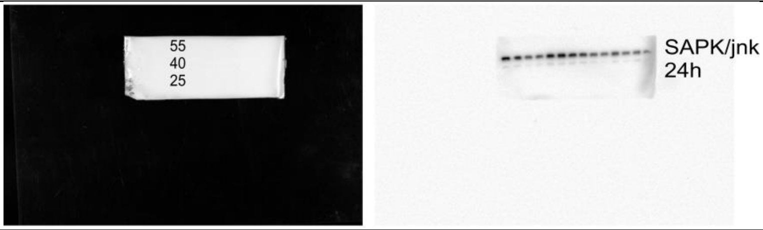
	GAPDH	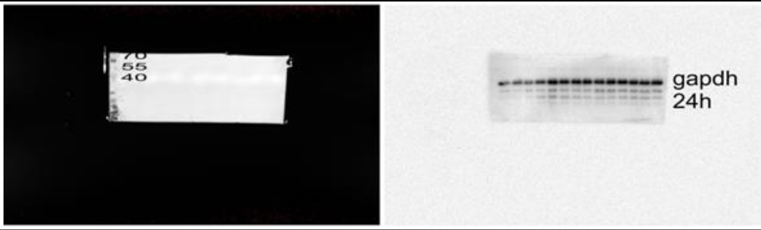
	pSAPK/JNK	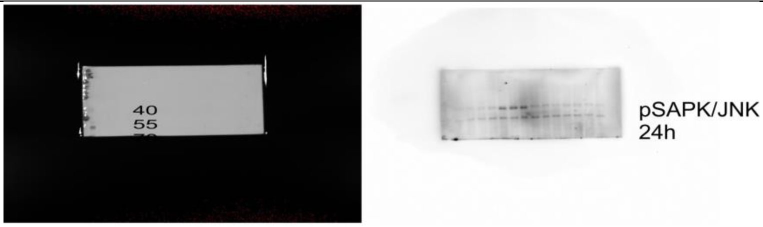

	GAPDH	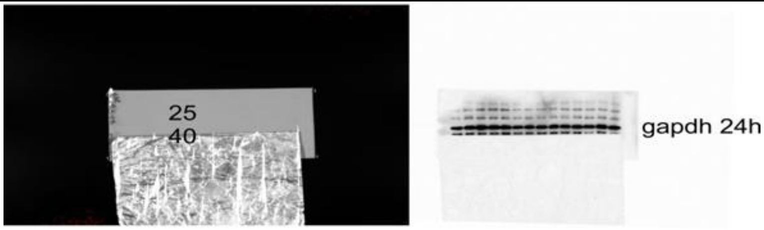
48h		
	SAPK/JNK	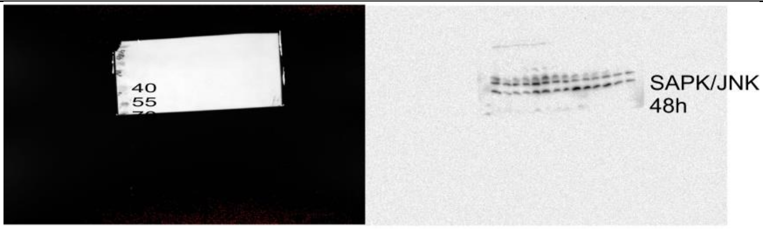
	pSAPK/JNK	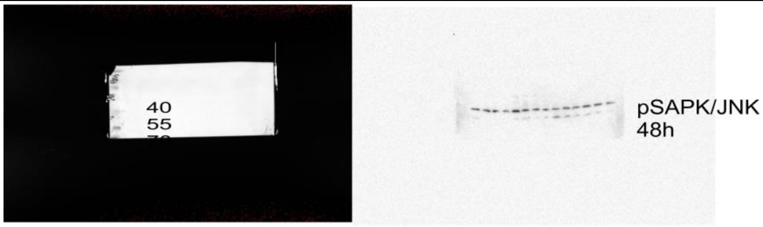
	GAPDH	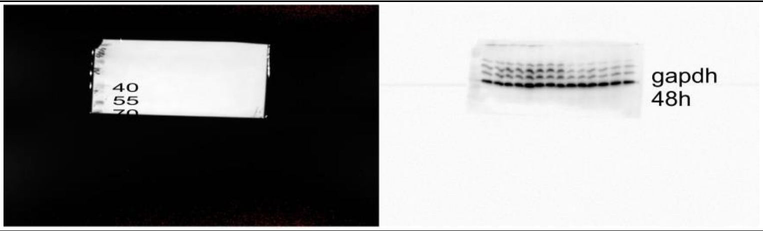
72h		
	SAPK/JNK	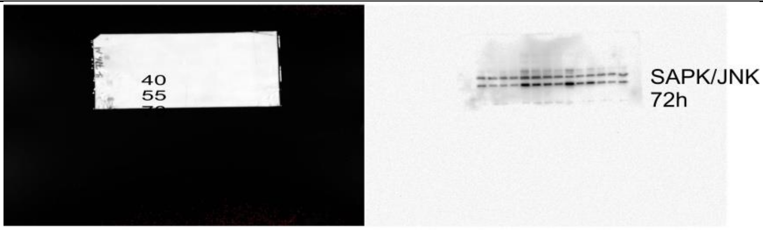
	pSAPK/JNK	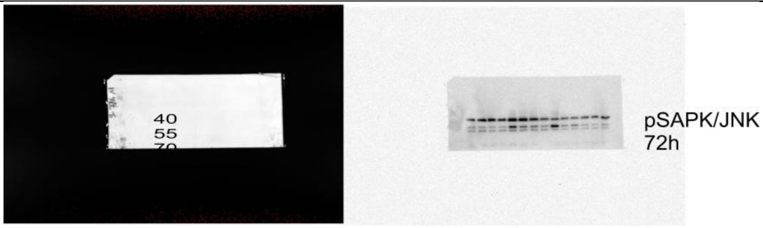
	GAPDH	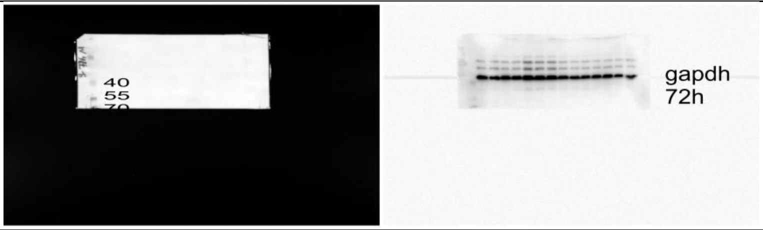

5- IkBα

<table>
<tr><td>2h</td><td></td><td></td></tr>
<tr><td></td><td>IkBα</td><td>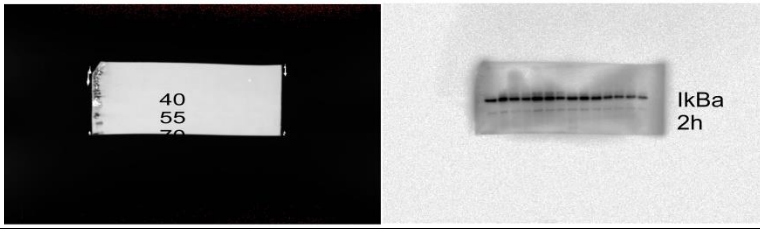</td></tr>
<tr><td></td><td>GAPDH</td><td>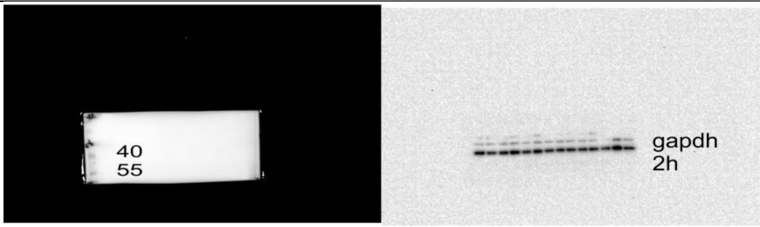</td></tr>
<tr><td></td><td>pIkBα</td><td>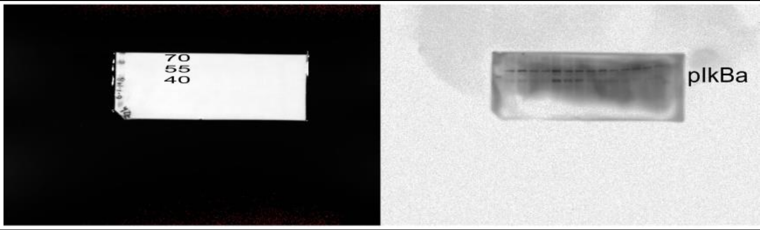</td></tr>
<tr><td></td><td>GAPDH</td><td>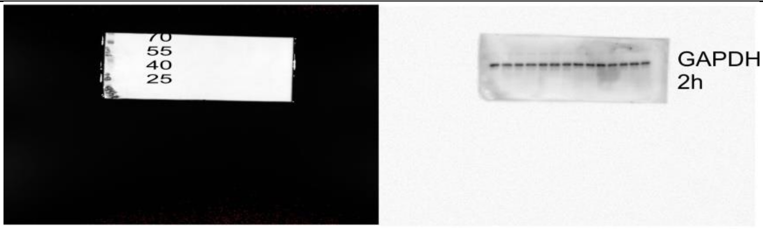</td></tr>
<tr><td>24h</td><td></td><td></td></tr>
<tr><td></td><td>IkBα</td><td>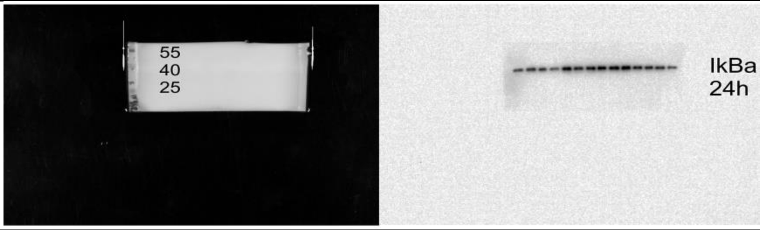</td></tr>
<tr><td></td><td>GAPDH</td><td>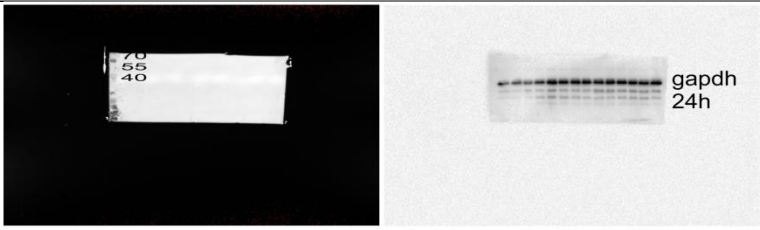</td></tr>
<tr><td></td><td>pIkBα</td><td>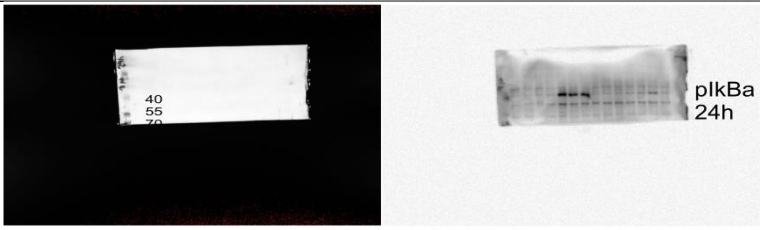</td></tr>
</table>

	GAPDH	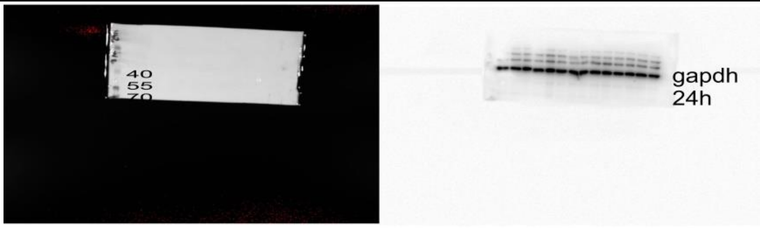
48h		
	IkBα	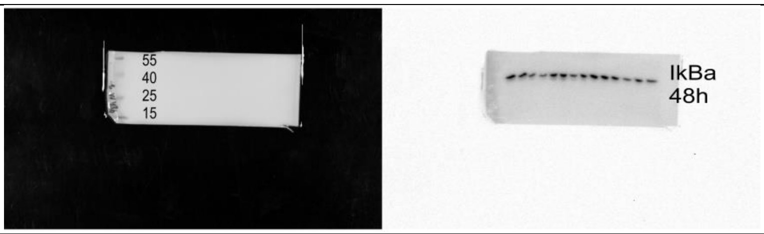
	GAPDH	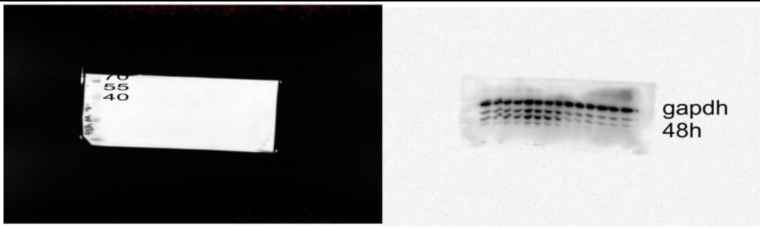
	pIkBα	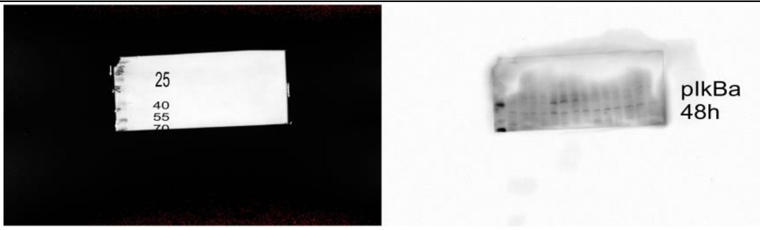
	GAPDH	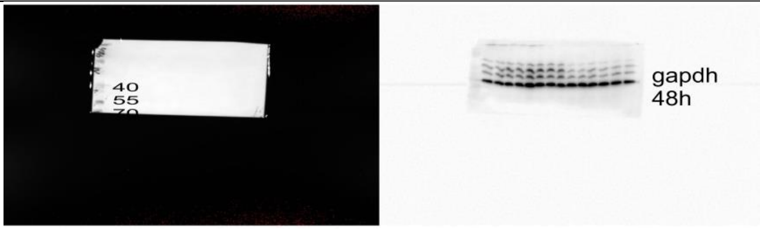
72h		
	IkBα	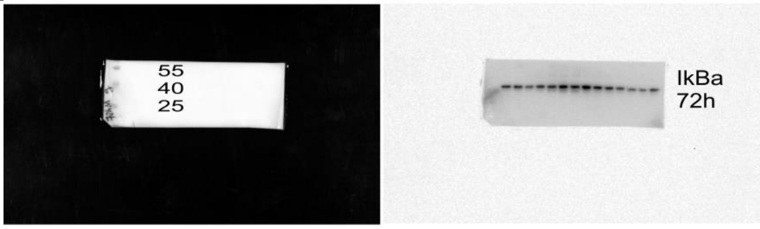
	GAPDH	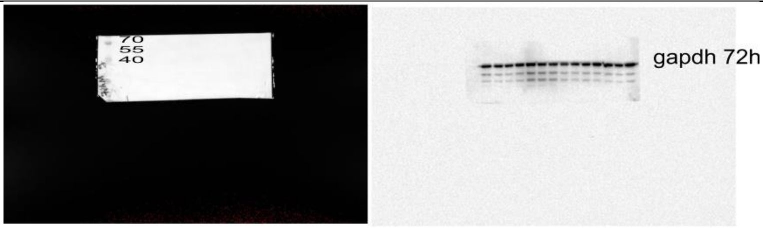

	pIkBα	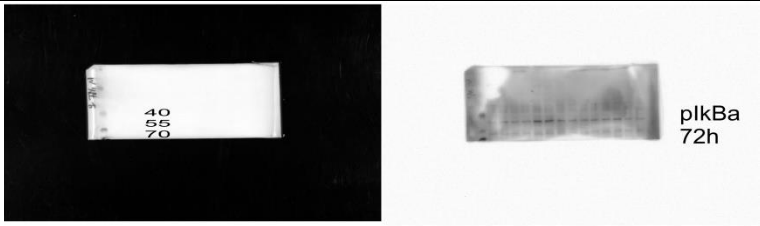
	GAPDH	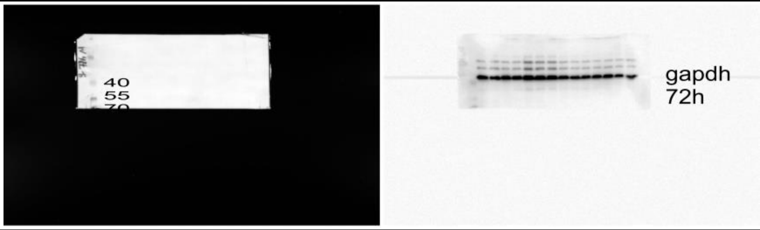

6- AKT

2h		
	AKT	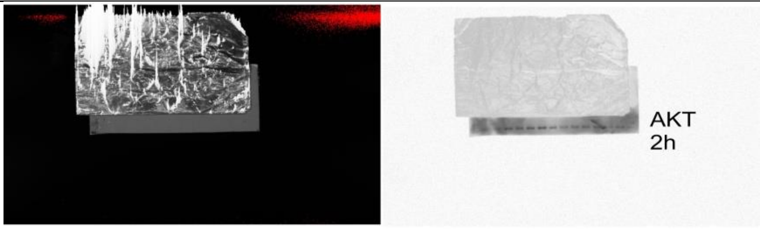
	GAPDH	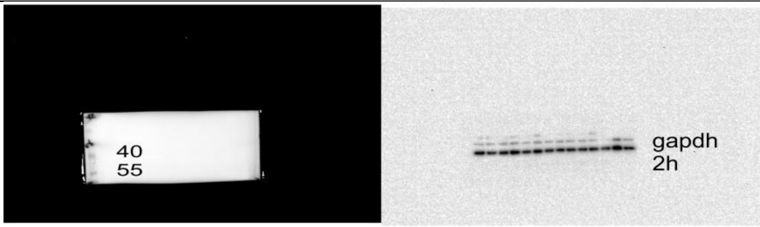
	pAKT	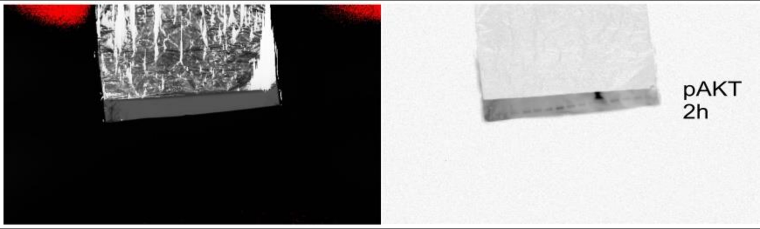
	GAPDH	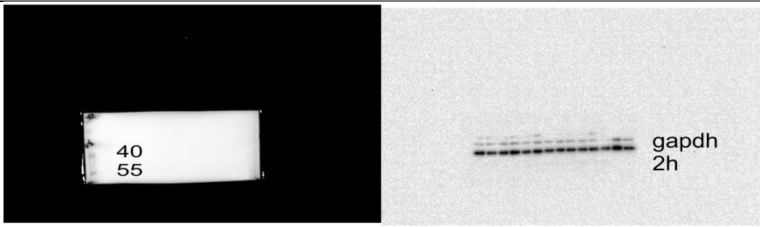
24h		
	AKT	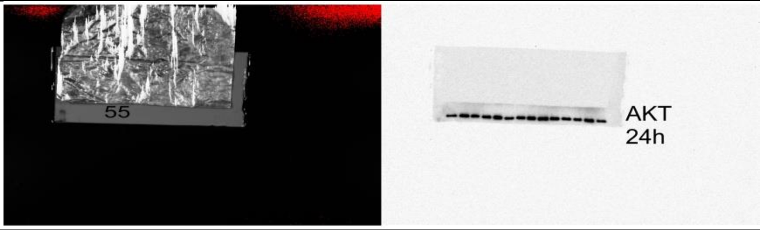
	GAPDH	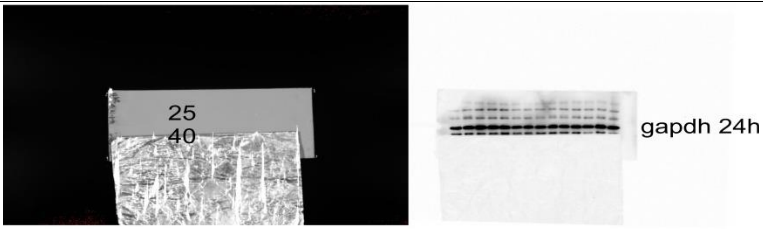
	pAKT	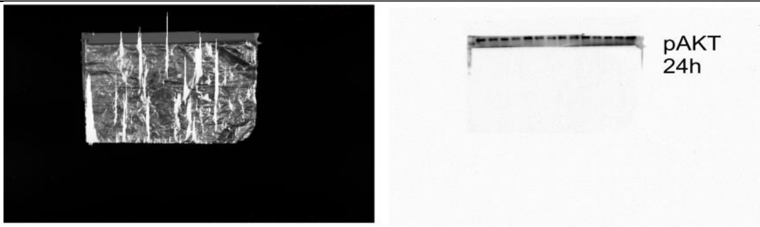

	GAPDH	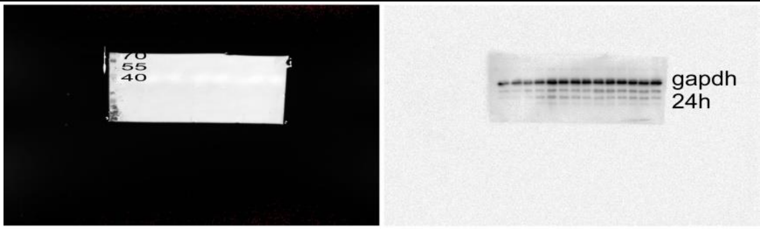
48h		
	AKT	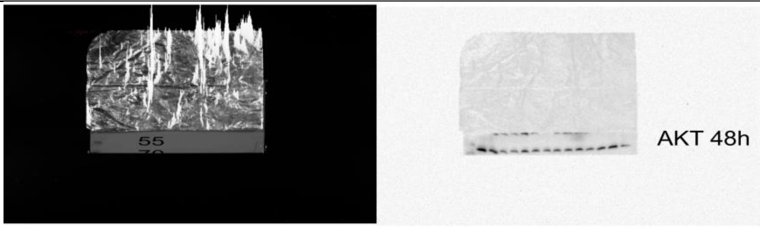
	GAPDH	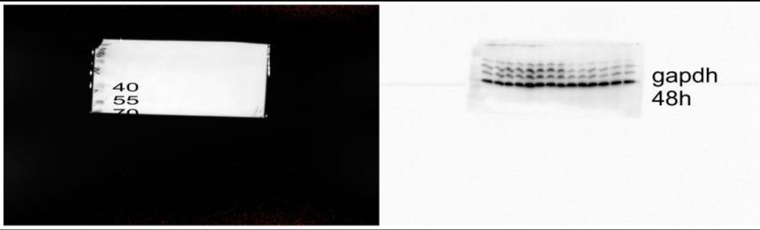
	pAKT	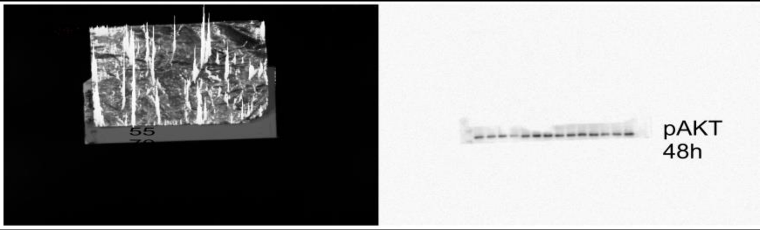
	GAPDH	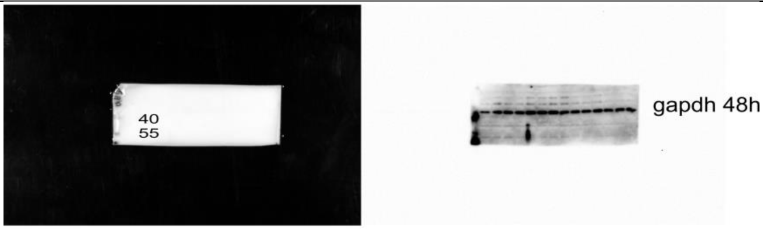
72h		
	AKT	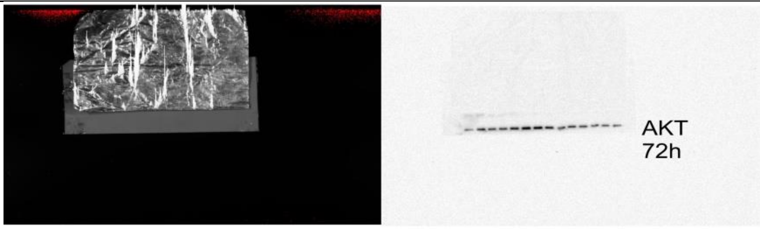
	GAPDH	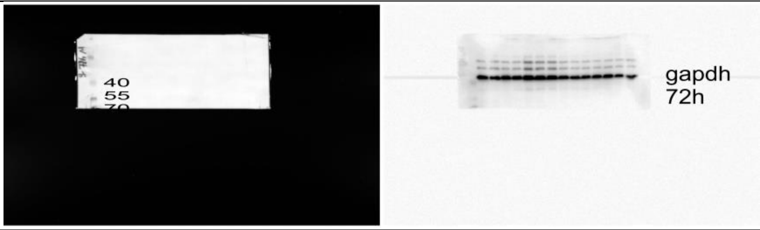

	pAKT	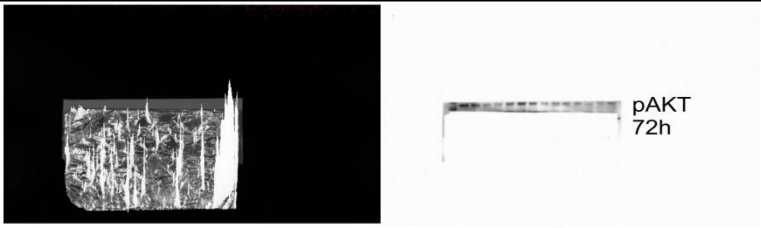
	GAPDH	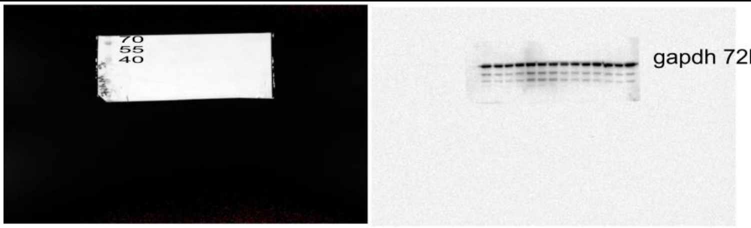

7- mTOR

2h		
	mTOR	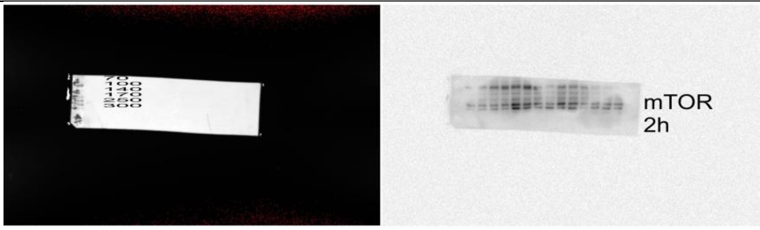
	GAPDH	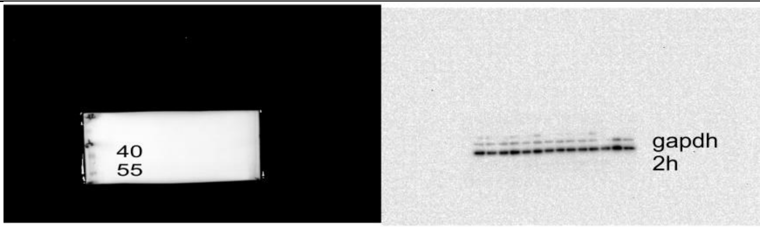
	pmTOR	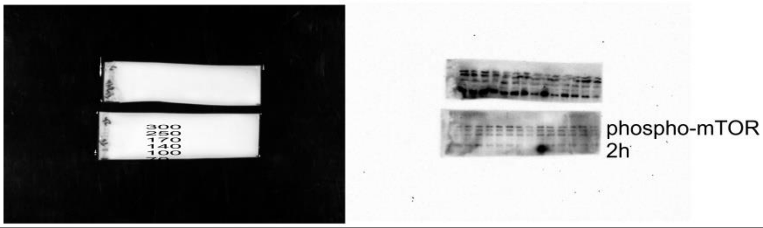
	GAPDH	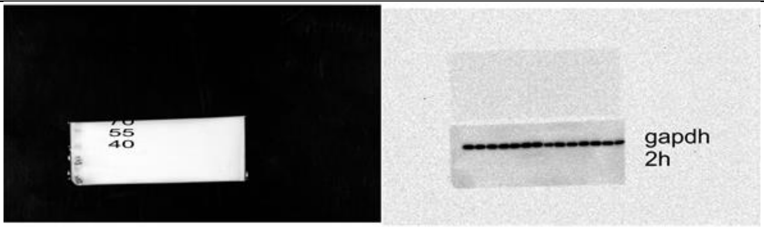
24h		
	mTOR	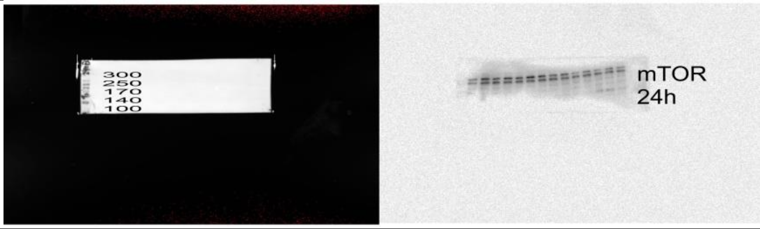
	GAPDH	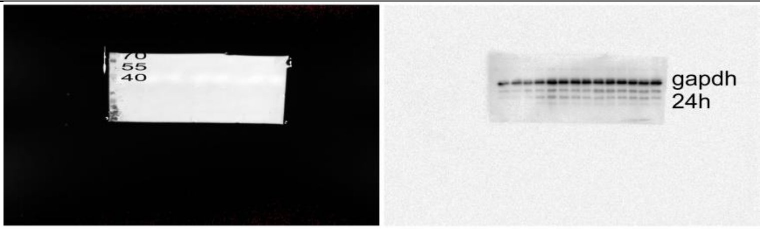
	pmTOR	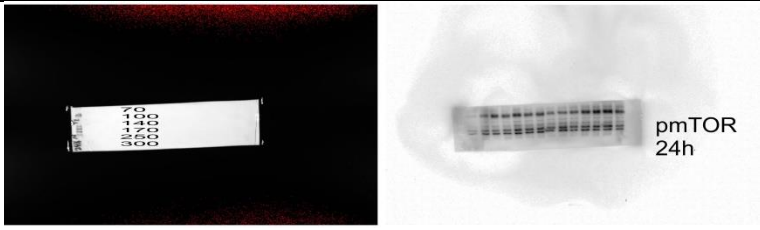

	GAPDH	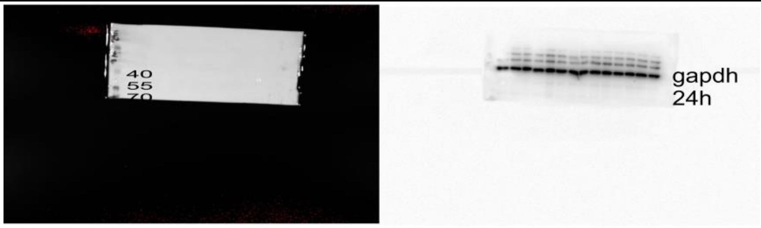
48h		
	mTOR	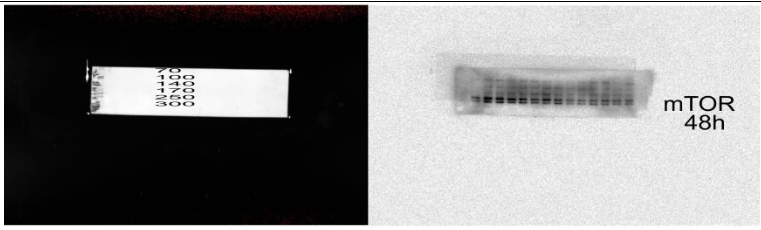
	GAPDH	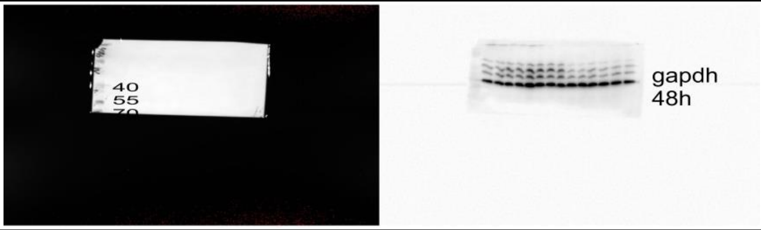
	pmTOR	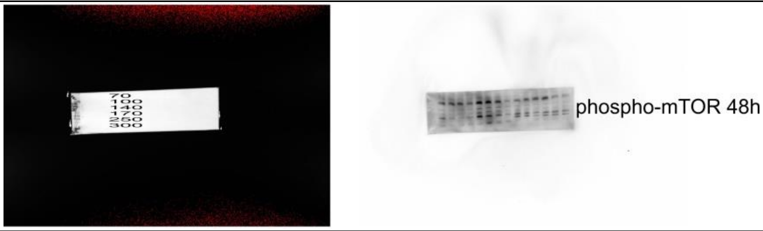
	GAPDH	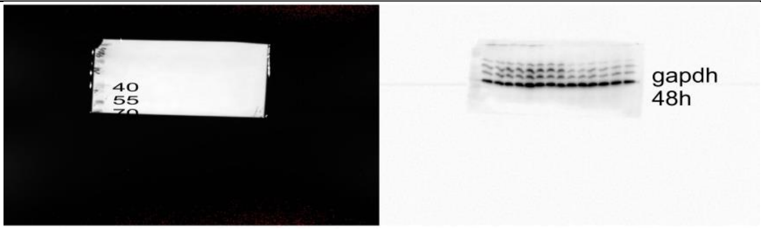
72h		
	mTOR	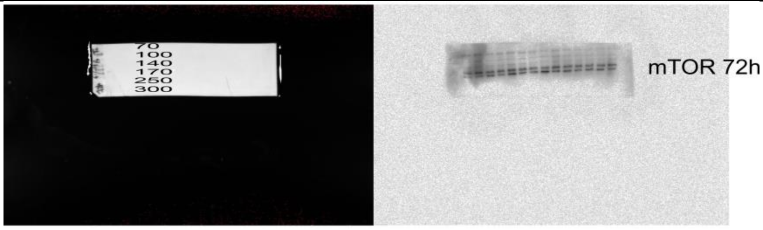
	GAPDH	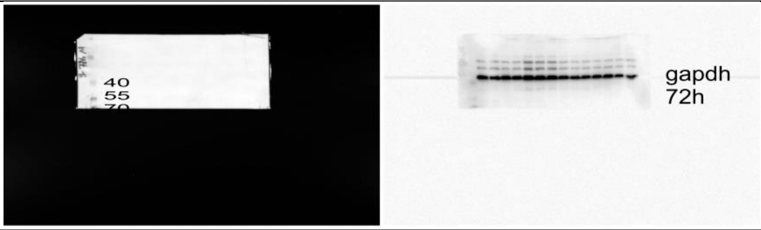

	pmTOR	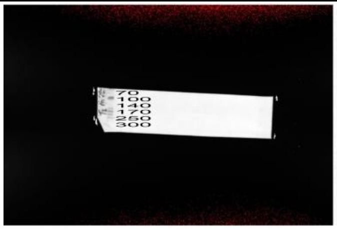 70<br>100<br>140<br>170<br>250<br>300 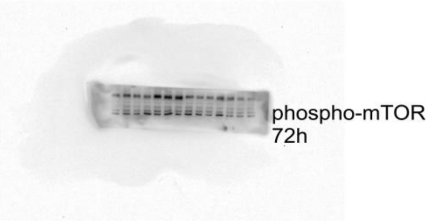 phospho-mTOR<br>72h
	GAPDH	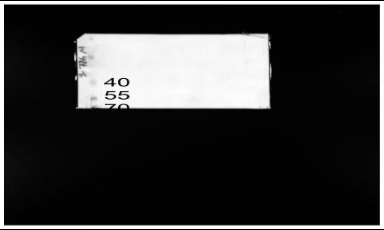 40<br>55 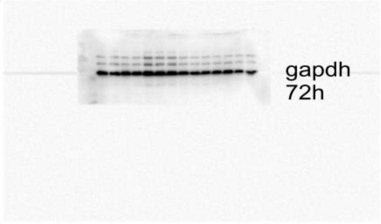 gapdh<br>72h

B

1. TLR4

TLR4	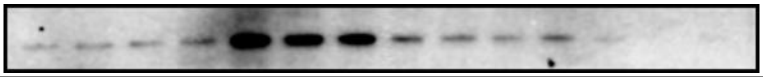	72h
GAPDH	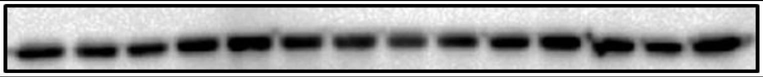	

2. ERK1/2

ERK1/2	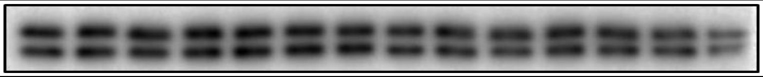	2h
GAPDH	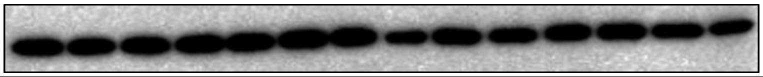	
pERK1/2	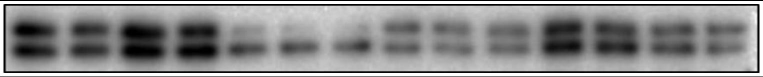	
GAPDH	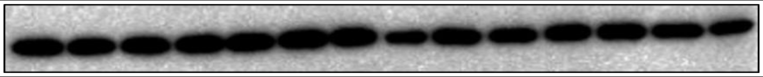	
ERK1/2	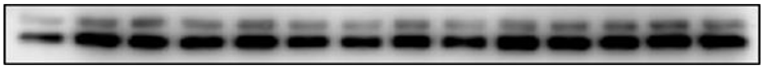	24h
GAPDH	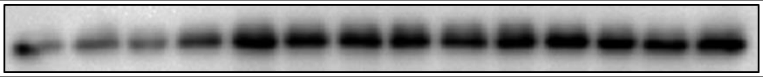	
pERK1/2	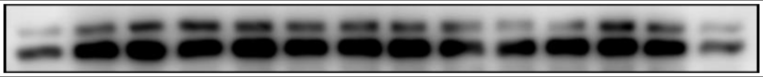	
GAPDH	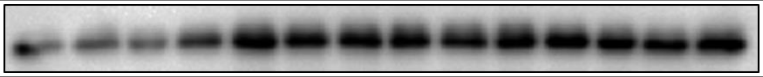	
ERK1/2	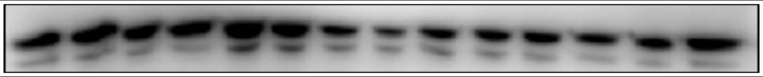	48h
GAPDH	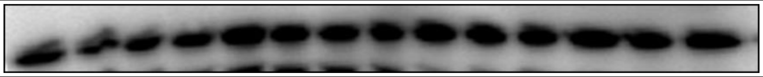	
pERK1/2	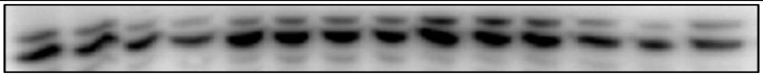	
GAPDH	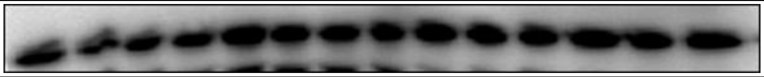	
ERK1/2	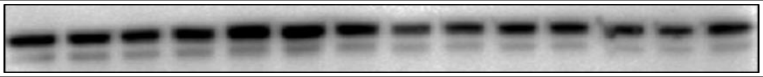	72h
GAPDH	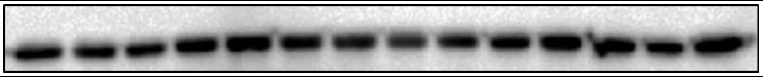	
pERK1/2	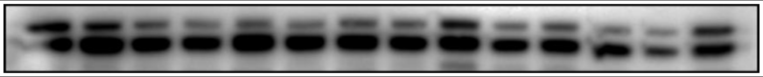	
GAPDH	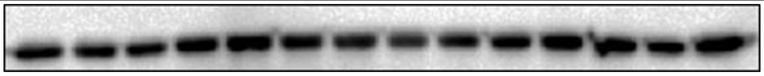	

3. p38

p38	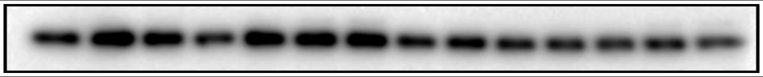	2h
GAPDH	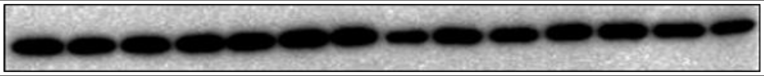	
pp38	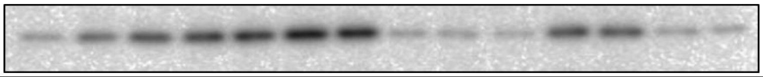	
GAPDH	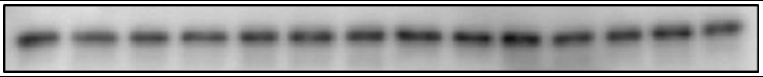	
p38	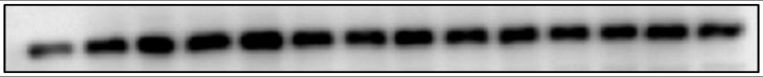	24h
GAPDH	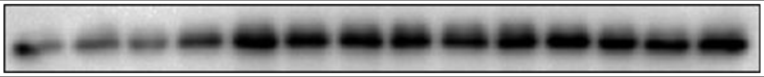	
pp38	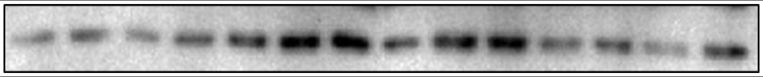	
GAPDH	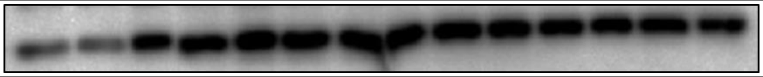	
p38	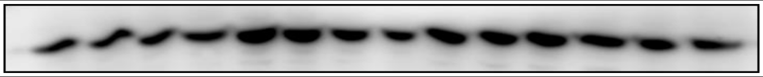	48h
GAPDH	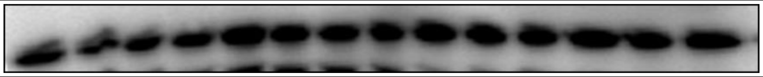	
pp38	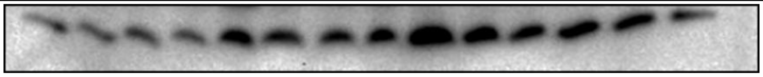	
GAPDH	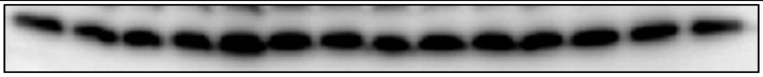	
p38	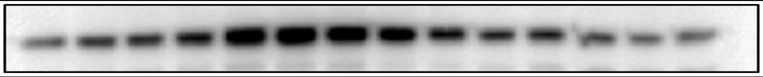	72h
GAPDH	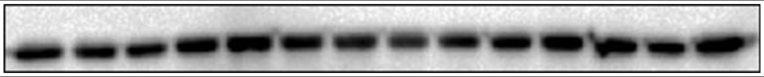	
pp38	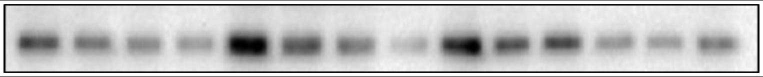	
GAPDH	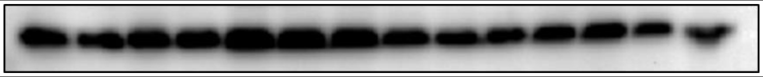	

4. SAPK/JNK

SAPK/JNK	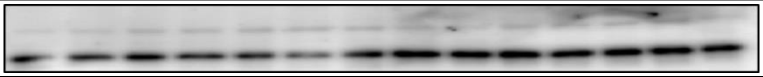	2h
GAPDH	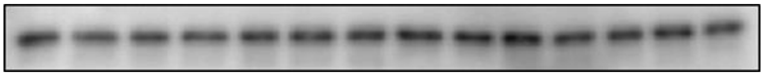	
pSAPK/JNK	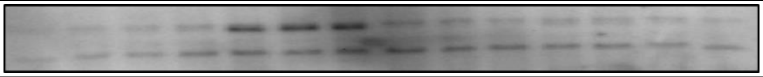	
GAPDH	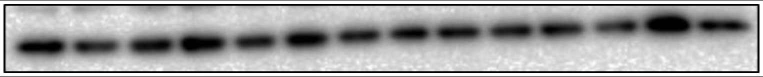	
SAPK/JNK	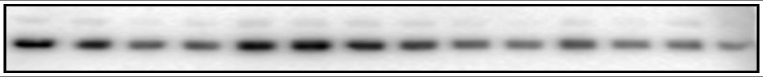	24h
GAPDH	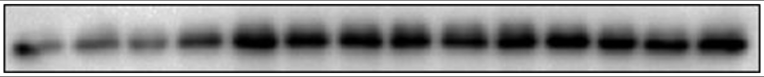	
pSAPK/JNK	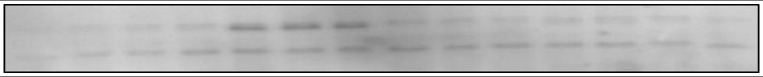	
GAPDH	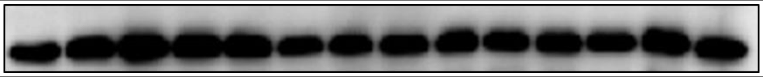	
SAPK/JNK	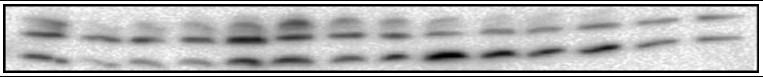	48h
GAPDH	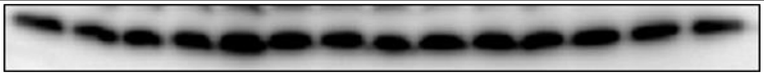	
pSAPK/JNK	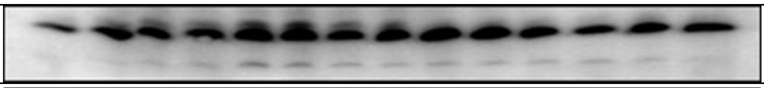	
GAPDH	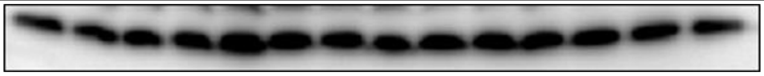	
SAPK/JNK	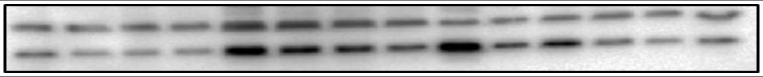	72h
GAPDH	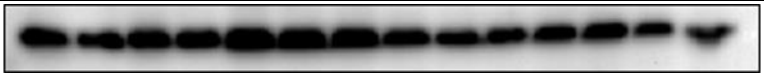	
pSAPK/JNK	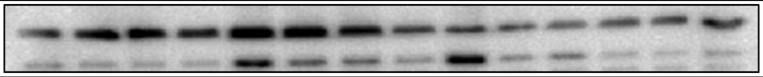	
GAPDH	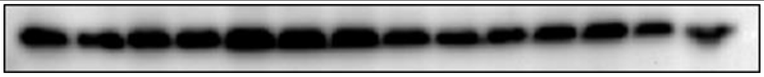	

5. IkBα

IkBα		2h
IkBα	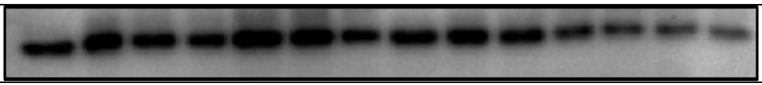	
GAPDH	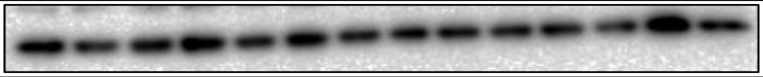	
pIkBα	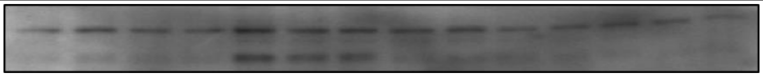	
GAPDH	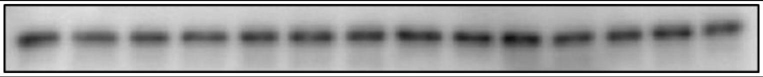	
IkBα	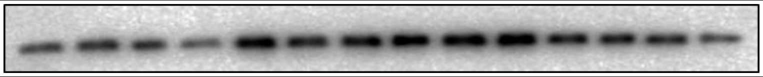	24h
GAPDH	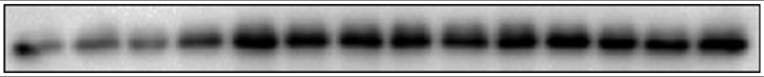	
pIkBα	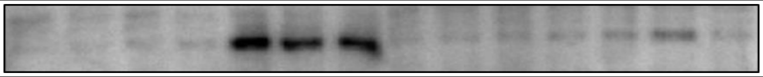	
GAPDH	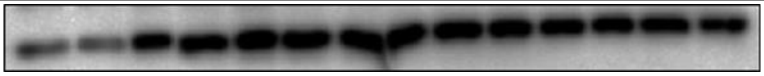	
IkBα	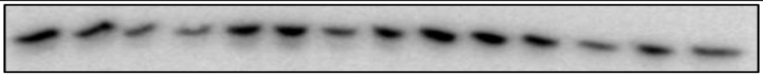	48h
GAPDH	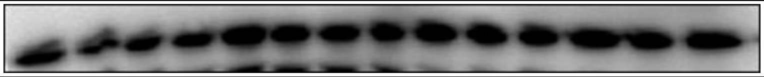	
pIkBα	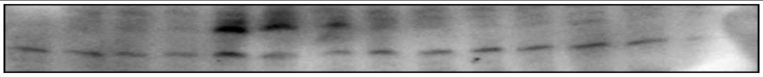	
GAPDH	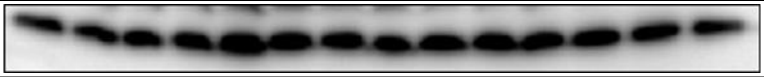	
IkBα	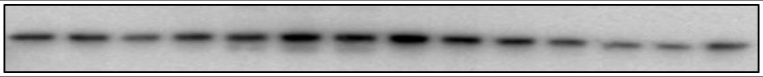	72h
GAPDH	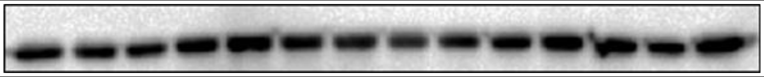	
pIkBα	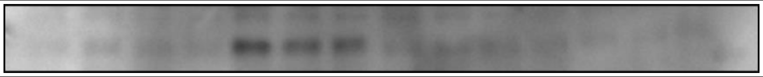	
GAPDH	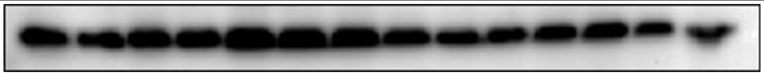	

6. AKT

**AKT**	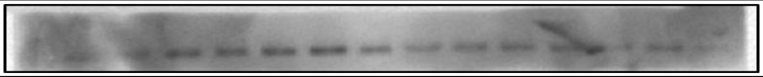	**2h**
**GAPDH**	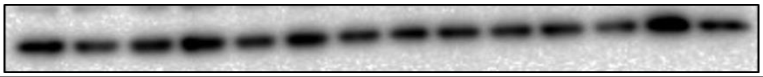	
**pAKT**	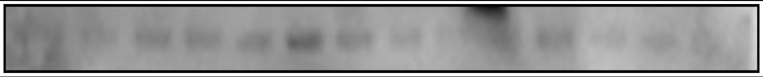	
**GAPDH**	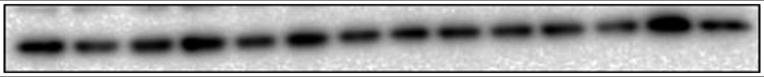	
**AKT**	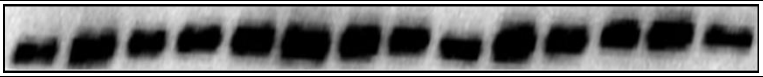	**24h**
**GAPDH**	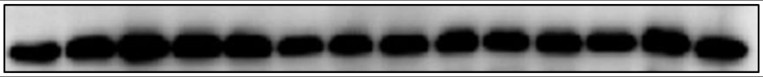	
**pAKT**	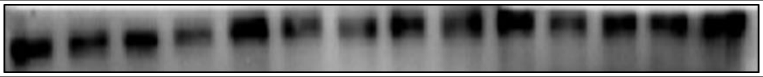	
**GAPDH**	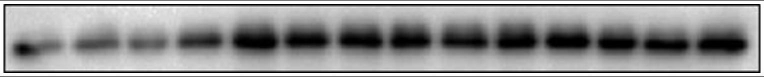	
**AKT**	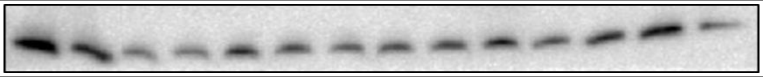	**48h**
**GAPDH**	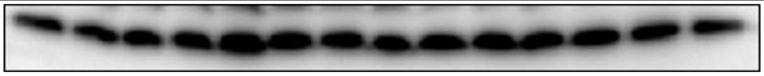	
**pAKT**	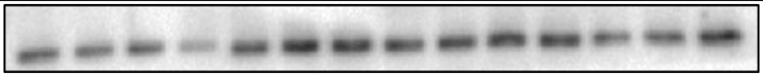	
**GAPDH**	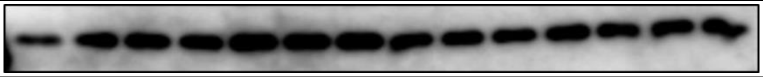	
**AKT**	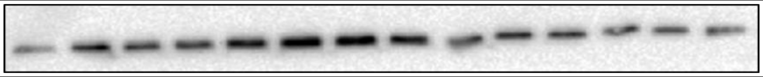	**72h**
**GAPDH**	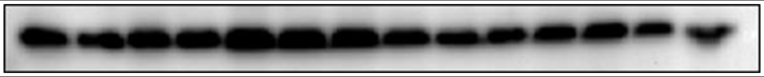	
**pAKT**	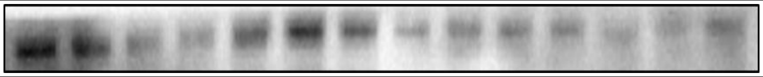	
**GAPDH**	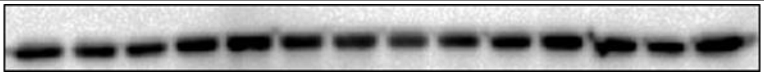	

7. mTOR

mTOR	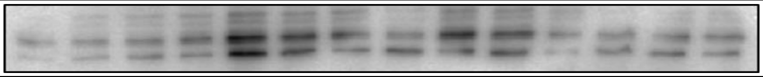	2h
GAPDH	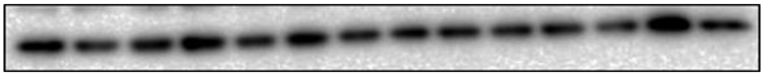	
Phopspho-mTOR	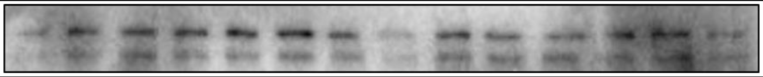	
GAPDH	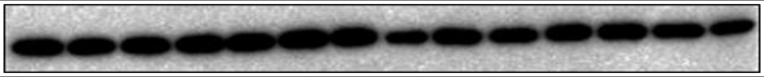	
mTOR	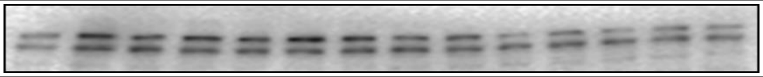	24h
GAPDH	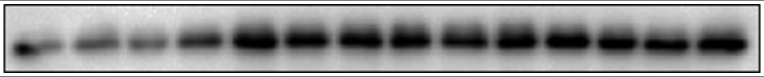	
Phopspho-mTOR	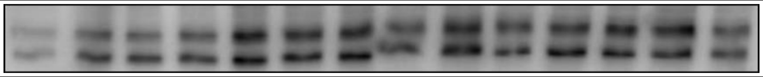	
GAPDH	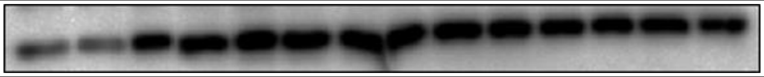	
mTOR	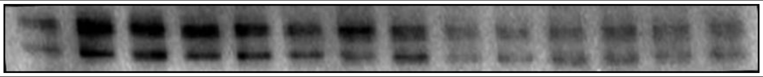	48h
GAPDH	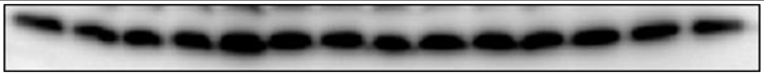	
Phopspho-mTOR	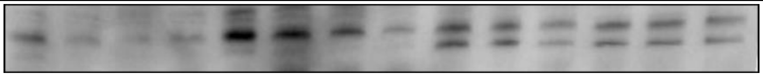	
GAPDH	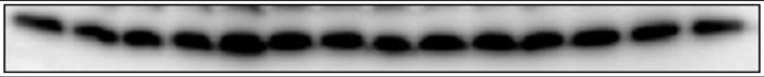	
mTOR	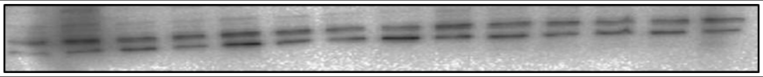	72h
GAPDH	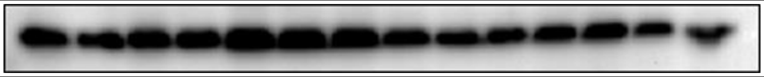	
Phopspho-mTOR	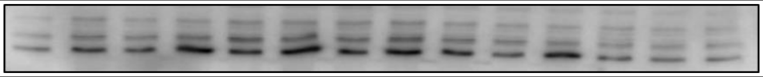	
GAPDH	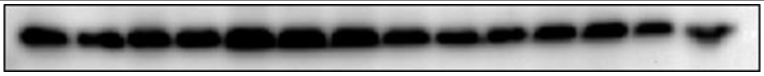	
